# The role of ADP-ribose metabolism in metabolic regulation, adipose tissue differentiation, and metabolism

**DOI:** 10.1101/gad.334284.119

**Published:** 2020-03-01

**Authors:** Magdolna Szántó, Peter Bai

**Affiliations:** 1 Department of Medical Chemistry, Faculty of Medicine, University of Debrecen, Debrecen 4032, Hungary;; 2 MTA-DE Lendület Laboratory of Cellular Metabolism, University of Debrecen, Debrecen 4032, Hungary;; 3 Research Center for Molecular Medicine, Faculty of Medicine, University of Debrecen, Debrecen 4032, Hungary

**Keywords:** PARP, ARTD, adipocyte, adipogenesis, mitochondria, lipolysis, differentiation, white adipocytes, brown adipocytes, beige adipocytes, stem cell, PARylation, high fat diet, obesity, insulin resistance, AFLD, NAFLD, atherosclerosis

## Abstract

Poly(ADP-ribose) polymerases (PARPs or ARTDs), originally described as DNA repair factors, have metabolic regulatory roles. PARP1, PARP2, PARP7, PARP10, and PARP14 regulate central and peripheral carbohydrate and lipid metabolism and often channel pathological disruptive metabolic signals. PARP1 and PARP2 are crucial for adipocyte differentiation, including the commitment toward white, brown, or beige adipose tissue lineages, as well as the regulation of lipid accumulation. Through regulating adipocyte function and organismal energy balance, PARPs play a role in obesity and the consequences of obesity. These findings can be translated into humans, as evidenced by studies on identical twins and SNPs affecting PARP activity.

## Abstract (precis)

In this review, Szanto et al. summarize the metabolic regulatory roles of PARP enzymes and their associated pathologies.

## Brief introduction to ADP-ribose metabolism

The field of poly(ADP-ribose) polymerases (PARPs or ARTDs) has come a long way since the discovery of a nuclear poly(ADP-ribosyl)ating (PARylating) enzyme in 1963 (Chambon et al. 1963). PARPs now constitute a superfamily of at least 17 members in human that share a conserved catalytic domain (Amé et al. 2004; Hottiger et al. 2010). ADP-ribosylation is a posttranslational modification, during which the ADP-ribosylation enzymes cleave NAD^+^ and attach the resulting ADP-ribose (ADPR) units to acceptor proteins. ADP-ribosylation is referred to as mono(ADP-ribosyl)ation (MARylation), oligo(ADP-ribosyl)ation, or poly(ADP-ribosyl)ation (PARylation), based on the number of the ADPR units added to the acceptor protein (Amé et al. 2004; Hottiger et al. 2010). Although all PARPs inherited the family name of the founding member, PARP-1, the PARP “polyenzymes” include only PARP-1, PARP-2, and the tankyrases (PARP-5a and PARP-5b) (Gibson and Kraus 2012). Other members perform only MARylation or oligo(ADP-ribosyl)ation, while PARP13 possesses no enzymatic activity (Hottiger et al. 2010). To our current understanding, the majority of PARP activity is attributed to PARP1 (80%–85%), while the rest is largely attributed to PARP2 (Amé et al. 1999; Schreiber et al. 2002; Szanto et al. 2011). In most cases, the major acceptor of PAR is PARP1 itself (termed auto-PARylation); nevertheless, with the use of state-of-the-art proteomics a large set of PARylated or ADP-ribosylated proteins were identified and this process is termed trans-PARylation (Chapman et al. 2013; Gibson et al. 2016; Abplanalp et al. 2018; Leslie Pedrioli et al. 2018; Palazzo et al. 2018) (for a comprehensive database of ADP-ribosylated proteins see Vivelo et al. (2017).

ADP-ribose unit(s) have rapid turnover and are removed by isoforms of poly(ADP-ribose) glycohydrolase (PARG) (O'Sullivan et al. 2019; Slade 2020), ADP-ribosyl hydrolase 3 (ARH3) (Oka et al. 2006; Rack et al. 2020), and ADP-ribosyl protein lyase (Kawaichi et al. 1983). PAR polymers can be recognized by a set of proteins that consequently localize to sites marked by PARP enzymes (Barkauskaite et al. 2013; Feijs et al. 2013). Karlberg et al. (2013) classified enzymes involved in ADPR metabolism and recognition as writers, readers, and erasers.

PARP1, PARP2, and PARP3 can be activated by DNA strand breaks and aberrant DNA forms (Menissier-de Murcia et al. 1989; Gradwohl et al. 1990; Kutuzov et al. 2013, 2015). Recently, other regulatory routes were described. PARP2 is activated by RNA forms (Léger et al. 2014); numerous signal transduction pathways, or the stability of PARP proteins were shown to modify the activity of PARP isoforms (Gagné et al. 2009; Cantó et al. 2013). PARPs, especially PARP1 and PARP2, are major NAD^+^ consumers in the cell (Bai et al. 2011a,b; Mohamed et al. 2014) and play a crucial role in regulating NAD^+^ availability and the nonredox functions of NAD^+^ (often referred to as the NAD^+^ node) (Houtkooper et al. 2010). On the other hand, PARP activity is dependent on NAD^+^ levels in cellular compartments and requires a continuous supply of NAD^+^. Nicotinamide mononucleotide adenylyl transferase (NMNAT) -1, -2, and -3 are NAD^+^ synthase enzymes that produce NAD^+^ from nicotinamide mononucleotide and ATP (Chiarugi et al. 2012; Cohen 2020). Thus, NMNATs can “feed” PARPs with their substrate and modulate PARP catalytic activity (Berger et al. 2007; Zhang et al. 2012; Ryu et al. 2018). There are pharmacological inhibitors available for the study of PARP biology, as well as for clinical use. Clinically available PARP inhibitors include ABT-888 (Veliparib from Abbott/Abbvie) rucaparib (Rubraca from Agouron/Pfizer/Clovis), talazoparib (Talzenna from Lead/Biomarin/Medivation/Pfizer), olaparib (Lynparza from KuDos Pharmaceuticals/AstraZeneca+Merck), and niraparib (Zejula from Merck/Tesaro/GSK) (for detailed review, see Slade 2020; Curtin and Szabo 2013). Although, none of the current PARP inhibitors seem to discriminate between PARP enzymes (Wahlberg et al. 2012), enzyme-specific inhibition of mono-PARP enzymes may be possible (Venkannagari et al. 2016; Upton et al. 2017; Holechek et al. 2018).

PARP enzymes have widespread biological functions ranging from DNA repair and chromatin structure (Javle and Curtin 2011; De Vos et al. 2012; Dantzer and Santoro 2013), RNA transcription, protein translation, and degradation (Kraus and Hottiger 2013; Bai 2015), cell division, tumor biology (Curtin and Szabo 2013), immune processes (Fehr et al. 2020) metabolism, and mitochondrial biology (Bai and Cantó 2012; Bai et al. 2015), oxidative stress biology, and cell death and differentiation, and aging (Mangerich et al. 2010; Burkle and Virag 2013; Fatokun et al. 2014). In this review, we focus on the metabolic properties of PARP enzymes.

## PARP enzymes in metabolism

PARP enzymes impact metabolism at multiple points, exerting regulatory functions on higher order organismal and basic cellular processes. From another perspective, PARPs impact both central and peripheral metabolic regulation. Frequently, PARP activation represent pathological disruptive metabolic signals. Here, we briefly review PARP-mediated pathways in metabolic regulation. Metabolic pathologies associated with PARP activation are listed in Table 1.

**Table 1.**
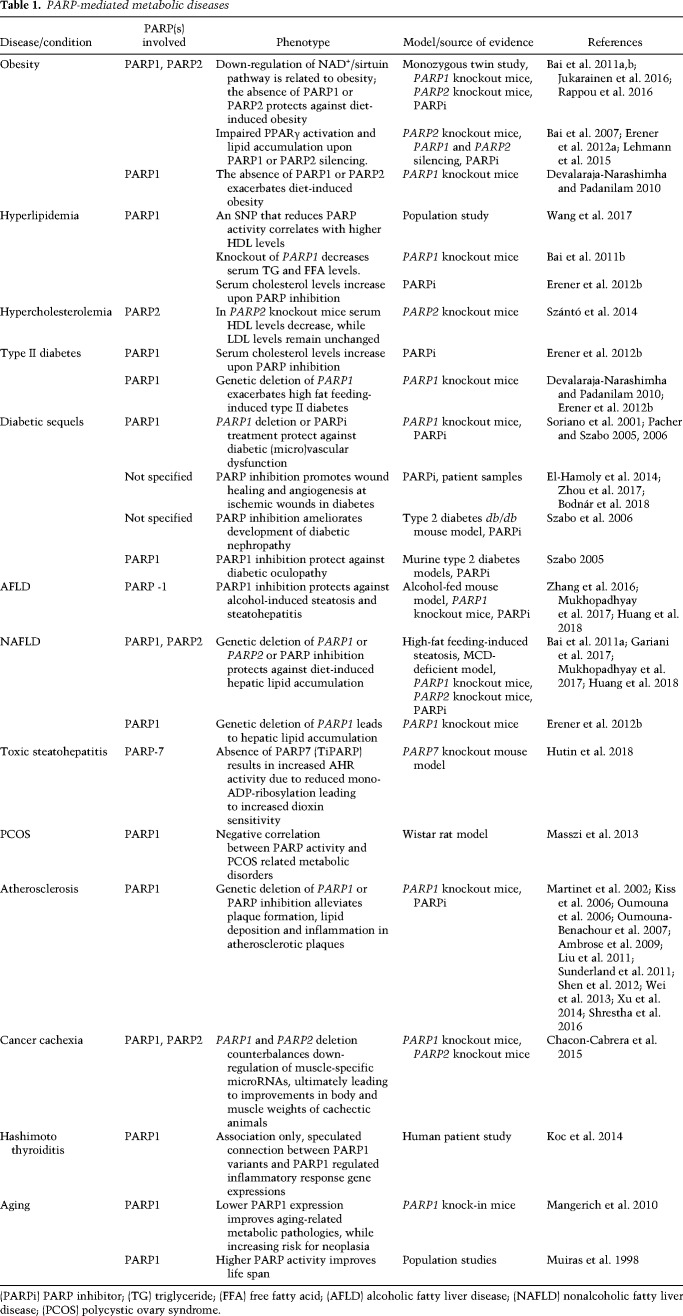
PARP-mediated metabolic diseases

### PARPs in regulating central and peripheral organismal metabolic homeostasis

PARP enzymes are widely expressed in almost all tissues and cells of the human organism, including metabolic tissues and organs, such as the liver, skeletal muscle, hormone glands, adipose tissue (white, brown, and beige), and the nerve system (Bai 2015). Central metabolic regulation encompasses the coordinated regulatory activity of the central nervous system and the hormonal system, which allows the organism to adjust to environmental and internal metabolic challenges. Such signals are integrated into the nuclei of the ventromedial hypothalamus, which serve as a central orchestrator and *zeitgeber* for other organs through hypothalamic neurohormonal changes (Cedernaes et al. 2019). Whole body genetic deletion of *PARP1* alters feeding entrainment in mice and changes spontaneous locomotor activity (Bai et al. 2011b), suggesting a role for PARP1 in the circadian phase of entrainment. PARP1 expression and PARP1 activity show circadian changes in murine models and humans that contribute to circadian entrainment of transcriptional programs in skeletal muscle, the liver, and in the cells of the immune system (Mocchegiani et al. 2003, 2004; Asher et al. 2010; Zhao et al. 2015). PARP1 can achieve circadian regulation of gene transcription through the following actions: (1) interacting with 11-zinc-finger protein or CCCTC-binding factor (CTCF) and converting parts of the chromatin to heterochromatin in a time-dependent fashion (Zhao et al. 2015) and (2) interacting with and ADP-ribosylating Clock protein (Asher et al. 2010). Yet-uncovered pathways may also be active. PARP1 activation seems to be vital for sensing or mediating NAD^+^/NADH levels to be integrated into cellular energy sensing and signaling. Although, the aforementioned pathways were described in nonneuronal models, PARPs are abundantly expressed and active in the nervous system (Komjati et al. 2004; Fatokun et al. 2014) and feeding and locomotion behavior changes in the *PARP1* knockout mice (Bai et al. 2011b), making it likely that these processes are active in neurons and other cellular elements of the nervous system. It is important to note that disrupting circadian entrainment increases the risk for obesity and the consequences of obesity (Kettner et al. 2015); however, this has not been studied in the context of PARP activation.

PARPs interfere with hormonal signaling at various points. PARPs regulate hormone levels, including intramuscular androgen production (Marton et al. 2018b). Fasting serum insulin levels were lower in *PARP2* knockout mice (Bai et al. 2011a), weak PARP inhibitors were shown to restore insulin expression (Ye et al. 2006) and the deletion of Tankyrase 1 (*PARP5a*, *TNK1*) induced serum insulin levels (Yeh et al. 2009). Pharmacological inhibition or genetic deletion of PARP1 protects against streptozotocin-induced β-cell death that impairs insulin production (Burkart et al. 1999). Interestingly, the deletion of *PARP2* impairs β-cell function and proliferation through blocking *pdx-1* (Bai et al. 2011a). PARP1 and PARP2 were shown to modulate adipokine expression (Bai et al. 2007; Yeh et al. 2009; Erener et al. 2012a,b; Lehmann et al. 2015).

The sensing of hormones is also regulated by PARPs. Nuclear hormone receptors use PARPs as cofactors (Table 2). Therefore, nuclear hormone receptor activation is PARP-dependent. Insulin-like growth factor (IGF)-1 signaling is potentiated by PARP inhibition (Amin et al. 2015). Furthermore, PARP1 interferes with GLP-1 signaling that may interfere with insulin secretion from β cells (Liu et al. 2011). PARP1 and PARP2 activation were shown to be a key step in the development of insulin resistance (for review, see Bai and Cantó 2012).

**Table 2.**
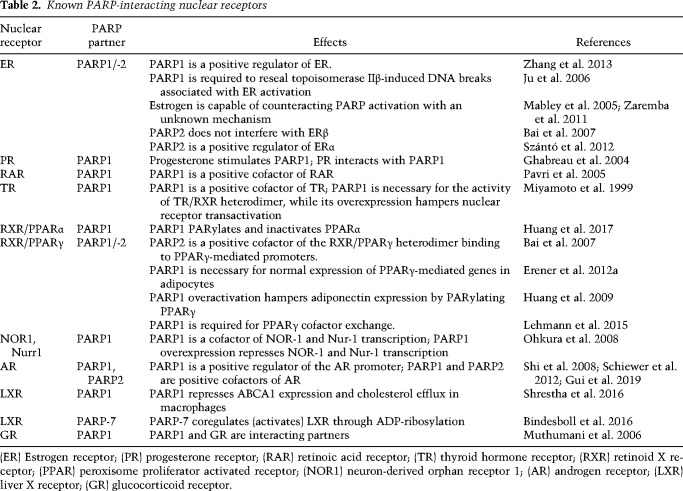
Known PARP-interacting nuclear receptors

Hormones, such as insulin (Horvath et al. 2008), estrogens (Mabley et al. 2005; Jog and Caricchio 2013; Joshi et al. 2014), androgens (Shimizu et al. 2013), progesterone (Ghabreau et al. 2004), artificial steroids, and vitamin D (Marton et al. 2018b) can modulate the expression and activity of PARP1 and PARP2. Endocrine disruptors were also shown to modulate PARP activity (Chen et al. 2013; Guerriero et al. 2018). These observations suggest feedback loops where PARPs interfere with hormonal signaling and hormones regulate PARP availability and activity.

PARPs interplay with energy sensor systems in cells (for review, see Bai et al. 2015). These systems assess the energy charge of cells (NAD^+^/NADH or ATP/(ADP + AMP) ratio) and the availability of nutrients (amino acids, oxygen, etc.) and shape cellular metabolism to meet these challenges.

### PARPs in carbohydrate metabolism

PARPs regulate points in glycolysis (Hopp et al. 2019), the core pathway of glucose catabolism. PARP1 activation hampers glycolytic flux, inducing metabolic dysfunction (Ying et al. 2002, 2003; Devalaraja-Narashimha and Padanilam 2009; Módis et al. 2012; Robaszkiewicz et al. 2014). Tankyrase 1 and Tankyrase 2 (TNK1, TNK2) regulate glucose transporter 4 (Glut4) translocation to the cytoplasmic surface in an ADP-ribosylation-dependent manner and, thus play a vital role in regulating glucose (and glutamine) availability and glycolytic flux (Yeh et al. 2007). The next step in glucose catabolism is the phosphorylation of glucose by hexokinase to form glucose-6-phosphate, which represents a commitment to glycolysis. Hexokinase is localized to the mitochondrial surface to help synchronize glycolytic flux and mitochondrial oxidation (Andrabi et al. 2014). PARP1 activation disrupts this synchronized function, reducing glycolytic influx (Andrabi et al. 2014; Fouquerel et al. 2014). This observation is further underlined by the observation that the supplementation of pyruvate, the end product of glycolysis, can alleviate cellular dysfunction and cell death upon PARP1 activation (Ying et al. 2002, 2003; Suh et al. 2005; Zeng et al. 2007). In agreement with these observations, the down-regulation of PARP1 supports glycolysis (Regdon et al. 2019). Glyceraldehyde-3-phosphate dehydrogenase (GAPDH) is an NAD^+^-dependent enzyme in glycolysis. PARP1 can PARylate and hence inhibit GAPDH (Du et al. 2003). Furthermore, since GAPDH is NAD^+^-dependent, NAD^+^ breakdown by cytoplasmic PARPs can limit GAPDH activity and, consequently, glycolytic flux (Hopp et al. 2019). These results were confirmed by the observation that *PARP1* knockout mice have higher respiratory quotient, suggesting a shift toward glucose oxidation (Bai et al. 2011b). Although pyruvate dehydrogenase complex is not considered as a member of the glycolytic enzymatic machinery, it is important to note that three subunits of the complex (PDPR, PDHA1, and PDHX) are subject to poly-ADP-ribosylation, which may regulate the fate of pyruvate, whether it can enter the TCA cycle, convert to lactate, or undergo gluconeogenesis (Hopp et al. 2019).

PARP10 and PARP14 are two poorly characterized members of the PARP family. Nevertheless, they seem to be connected to carbohydrate metabolism. Silencing of PARP10 induces glycolysis and mitochondrial oxidation, rendering cells hypermetabolic (Márton et al. 2018a). PARP14 can support glycolysis in lymphoma cells, although the molecular mechanism has not been elucidated (Cho et al. 2011). Another interesting feature of PARP14 is its physical interaction with phosphoglucose isomerase, an enzyme that enables the entry of fructose into glycolysis (Yanagawa et al. 2007). The actual consequence of this interaction is unknown.

When considering carbohydrate metabolism, the regulatory mechanisms should also be mentioned. PARPs interact with HIFs, GSK3b, and AMPK, sensors that regulate glycolytic flux and the coupling of glycolysis to mitochondrial oxidation. These pathways are reviewed in Bai et al. (2015). A high-glucose or high-fructose diet can induce the expression of PARP1 (Choi et al. 2017; Huang et al. 2019). The interplay between carbohydrate metabolism and PARPs was extensively reviewed in Hopp et al. (2019).

### PARPs in lipid metabolism

There is an ever-growing body of evidence for the involvement of PARPs in lipid metabolism. As a prime example, PARP2 was found to be connected to cholesterol and triglyceride metabolism in a genome-wide association study (Manunza et al. 2014).

Cellular and organismal fatty acid homeostasis are regulated by PARPs. Erener et al. (2012b) reported hypercholesterolaemia in *PARP1* knockout mice. The pattern of polyunsaturated fatty acid metabolites is dysregulated in *PARP1* knockout mice (Kiss et al. 2015) and there seems to be a correlation between PARP1 activity and erythrocyte membrane composition (Bianchi et al. 2016). Furthermore, the composition of membrane-constituent lipids was altered upon the deletion of *PARP2* (Marton et al. 2018b).

Fatty acid absorption and fatty acid biosynthesis had not been studied in the context of PARP enzymes and poly-ADP-ribosylation; however, the involvement of PARPs is likely, as suggested by scattered data in the literature. For example, the deletion of *PARP2* reduces the expression of fatty acid synthase in the white adipose tissue (Bai et al. 2007). The expression of the fatty acid transporters, FABP7, FABP3, CD36, and aP2 (FABP4), are regulated by PARP1, PARP2, and tankyrases (Bai et al. 2007; Yeh et al. 2009; Erener et al. 2012a; Kiss et al. 2015). The deletion of *PARP1*, *PARP2*, or *PARP10* induces mitochondrial fatty acid oxidation (Bai et al. 2011a,b; Márton et al. 2018a). Upon the genetic deletion of *PARP2*, the respiratory quotient decreases, suggesting a preference for fatty acid oxidation both in the active and in the sleeping period of the daily cycle (Bai et al. 2011a). Acylation of histone proteins by fatty acids may serve as epigenetic marks, a recent study suggested the PARP-sirtuin interplay may be a key factor in regulating acyl epigenetic marks (Faraone-Mennella et al. 2019).

Certain fatty acid-type lipid species can regulate the expression of PARPs. Serum deprivation of a plethora of lipid species (Sun et al. 2019) can inhibit PARP2 expression, similar to lipoic acid (Zhang et al. 2014). Caloric restriction reduces, while a high-fat diet induces the expression of PARP1 (Bai et al. 2011b; Salomone et al. 2017; Huang et al. 2019). In a similar fashion, fatty acid synthase activation or overexpression can also induce PARP1 expression (Wu et al. 2016).

Another arch of lipid metabolism is cholesterol homeostasis and the metabolism of cholesterol derivatives. The central organ for cholesterol biosynthesis is the liver, although other organs, such as skeletal muscle, also possess functional enzymatic machinery for cholesterol biosynthesis. Dietary cholesterol is taken up from the intestines and is then transported to the liver by chylomicrons. Excess cholesterol is excreted in the bile that is subsequently emptied into the intestines. Collectively, this is called the enterohepatic circulation of cholesterol. The liver can excrete cholesterol into low-density lipoprotein (LDL) that are then sent to the periphery to supply cholesterol to cells. Peripheral cholesterol is returned to the liver by high-density lipoproteins (HDL). This is the peripheral circulation of cholesterol in humans. Mice have little HDL, therefore, LDL performs the functions of HDL in mice. Cholesterol is a starting compound for the synthesis of steroid hormones, vitamin D, and bile acids.

PARP2 negatively regulates de novo cholesterol biosynthesis through suppression of sterol-regulatory element-binding protein expression. The deletion of PARP2 induces increased cholesterol biosynthesis in the liver and skeletal muscle (Szántó et al. 2014; Marton et al. 2018b). A fraction of excess cholesterol seems to be incorporated into biomembranes (Marton et al. 2018b). The deletion of *PARP2* does not affect the enterohepatic circulation of cholesterol. However, *PARP2* deletion reduces the expression of hepatic ATP-binding cassette subfamily A member 1 (ABCA1), a major transporter of cholesterol to lipoproteins (Szántó et al. 2014). In line with this, serum HDL levels are lower in *PARP2* knockout mice (Szántó et al. 2014). However, it is not easy to translate this finding into the human situation.

PARP1 expression and activity correlate negatively with ABCA1 expression (Shrestha et al. 2016). In addition, PARP1 regulates the expression of microsomal epoxide hydrolase (mEH), a key sodium-dependent bile acid transporter in hepatocytes (Peng et al. 2015). Furthermore, a lipid-activated enzyme, acyl-CoA-binding domain containing 3, activates PARP1 activity (Chen et al. 2015). Knockout and pharmacological inhibitor studies show that PARP1 inhibition improves HDL/LDL levels in mice (Diestel et al. 2003; Kiss et al. 2006; Oumouna-Benachour et al. 2007; Hans et al. 2008; von Lukowicz et al. 2008; Zerfaoui et al. 2008; Hans et al. 2009a, b; Xu et al. 2014). In humans, an SNP that renders PARP1 less active correlates with decreases total cholesterol levels, increases in HDL and decreased risk for coronary artery disease (Wang et al. 2017).

Lipids can be stored physiologically or pathophysiologically in multiple organs, where excess lipids cause damage to the tissue. Lipid-mediated activation of PARP1 may have a crucial role in organ or cellular damage (Diestel et al. 2003; Kiss et al. 2006; Hans et al. 2008; Bai and Csóka 2015; Chen et al. 2015). Ectopic lipid deposition to the walls of arteries happens in atherosclerosis. PARP inhibition or genetic deletion of *PARP1* alleviates the symptoms of atherosclerosis by reducing plaque area, lipid deposition, inflammation, and the HDL/LDL ratio (Martinet et al. 2002; Kiss et al. 2006; Ambrose et al. 2009; Liu et al. 2011; Sunderland et al. 2011; Shen et al. 2012; Wei et al. 2013; Xu et al. 2014).

The liver, although it has limited lipid storage, is also a site for abnormal lipid deposition in alcoholic and nonalcoholic fatty liver disease (AFLD and NAFLD, respectively). Alcohol consumption induces PARylation (Nomura et al. 2001). Logically, pharmacological PARP inhibition confers protection against steatosis, inflammation, and liver tissue injury in AFLD (Mukhopadhyay et al. 2017). While the genetic deletion of *PARP2* is protective against nonalcoholic hepatic lipid accumulation (Bai et al. 2011a), there is apparent ambiguity in the literature on the role of PARP1 concerning whether the genetic ablation of *PARP1* exacerbates NAFLD (Erener et al. 2012b) or pharmacological PARP inhibition protects against steatosis, inflammation, and liver tissue injury in NAFLD (Bai et al. 2011b; Gariani et al. 2017; Mukhopadhyay et al. 2017; Huang et al. 2018). The differences have not been elucidated yet.

## General outline of adipogenesis

“Professional” lipid storage cells in mammals are adipocytes classified as white, brown, and beige adipocytes.

Brown or multilocular (referring to the numerous intracellular lipid droplets) adipocytes are localized to specific regions, including the interscapular and perirenal regions and lining the large arteries (Cannon and Nedergaard 2004). Brown adipocytes are characterized by high mitochondrial content and high uncoupling protein-1 (UCP1) expression (Kajimura 2015). This tissue is vital in human newborns and in rodents for maintaining core body temperature through uncoupled respiration and through that, in maintaining organismal energy balance, regulating fatty acid and glucose oxidation, and preventing or alleviating obesity and its consequences (Cannon and Nedergaard 2004).

Beige adipocytes are localized within white adipose tissue depots mixed with white adipocytes (Wu et al. 2012). Beige cells share the morphological characteristics of white adipocytes; nevertheless, beige cells respond to adrenergic stimuli by mitochondrial biogenesis, induction of UCP1 expression, fatty acid breakdown, and heat generation. Beige adipocytes are characterized by a futile creatine cycle (Kristóf et al. 2016; Bertholet et al. 2017; Kazak et al. 2017) that is not present in brown cells and is vital for heat generation. Importantly, a mutation in the *fto* gene was associated with impaired beige adipogenesis and, consequently, impaired mitochondrial biogenesis and organismal energy balance (Claussnitzer et al. 2015).

White adipocytes are cells specialized for fat storage. Morphologically, these cells are unilocular and when stimulated respond with triglyceride breakdown through hormone-sensitive lipase (HSL). There are multiple adipose tissue depots in the body and their metabolic behavior is quite different in terms of lipid mobilizing capacity or heat generation (Garaulet et al. 2006; Roca-Rivada et al. 2011; Sacks et al. 2013; Luche et al. 2015). The switching on of beige adipocytes in white adipose depots or the transdifferentiation of white adipocytes to brown or beige cells is termed “browning” (Kajimura 2015).

**Figure 1.**
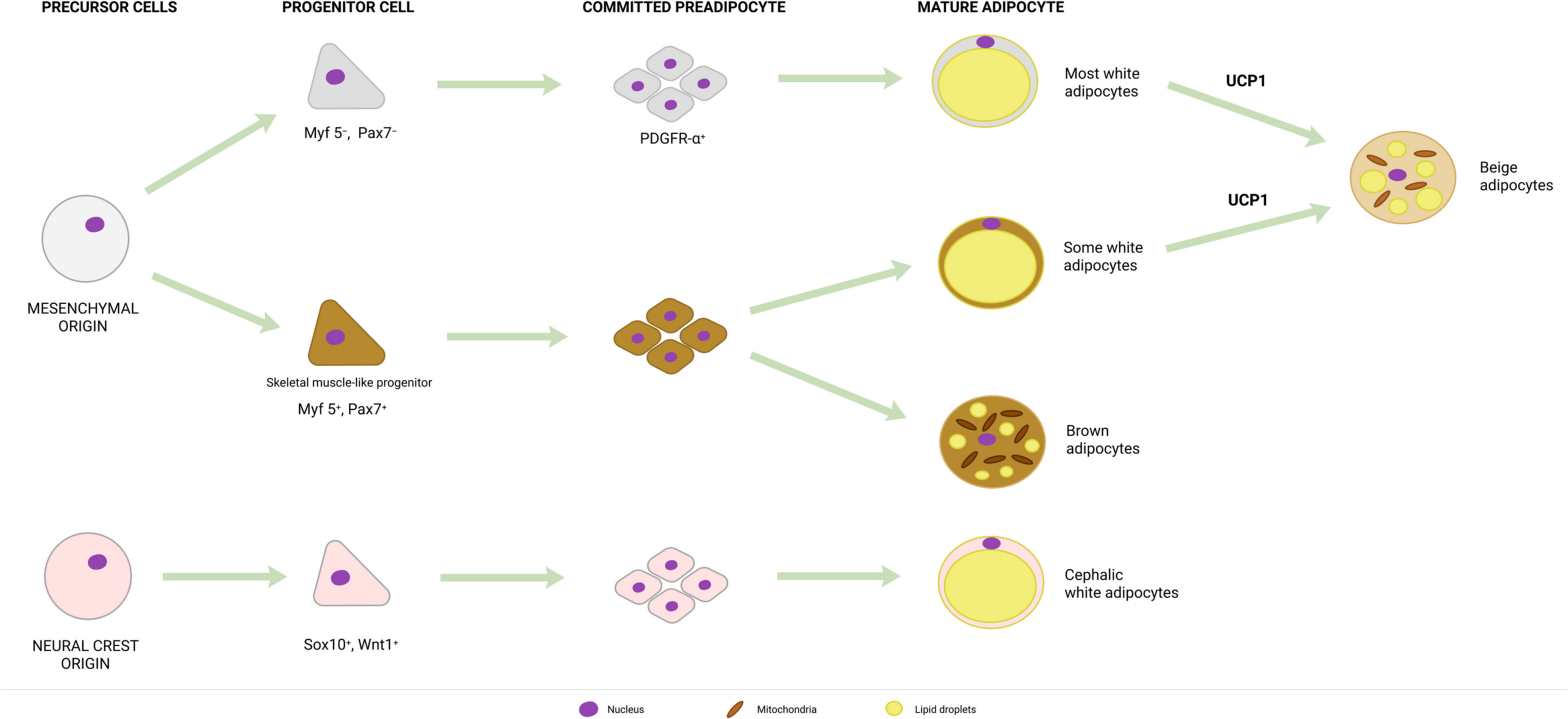
The general scheme of adipose tissue lineage differentiation. Abbreviations are defined in the text.

According to the classical scheme of adipocyte differentiation, *Pax7^+^ Myf5^+^* brown cell precursors segregate from the dermatomyotome, while *Pax7^−^ Myf5^−^* stem cells differentiate to white and beige adipocytes (Rosen and Spiegelman 2014). This picture is, in fact, more complex (Fig. 1). Lineage tracing studies revealed that there are multiple lineages giving rise to white adipocytes. The majority of these are of mesenchymal origin; nevertheless, depots in the head region stem from the neural crest (*Sox10^+^*, *Wnt1^+^* precursors) (Billon et al. 2007; Sanchez-Gurmaches and Guertin 2014a). Mesenchymal precursors can be *Myf5^+^* or *Myf5^−^*. The proportion of white adipocytes derived from *Myf5^+^* or *Myf5^−^* precursors vary between the adipose tissue depots (Sanchez-Gurmaches and Guertin 2014a). Beige adipocytes can differentiate from the same precursors as the white adipocytes, except for neural crest-derived precursors (Sanchez-Gurmaches and Guertin 2014a). Finally, brown adipocytes differentiate from *Pax7^+^ Myf5^+^* dermatomyotomal precursors (Sanchez-Gurmaches and Guertin 2014a).

**Figure 2.**
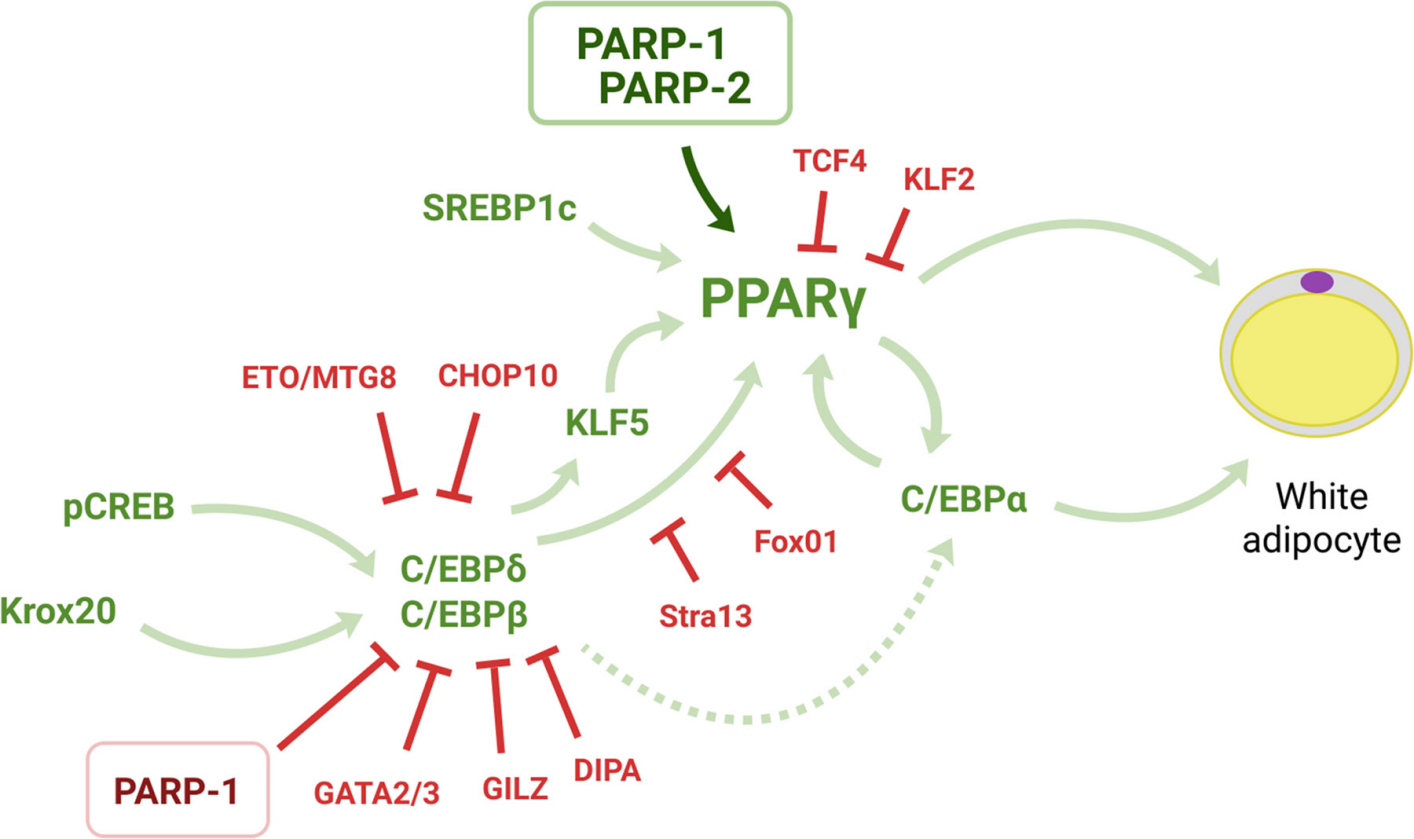
The involvement of PARP enzymes in the transcriptional control of white adipogenesis. Abbreviations are defined in the text.

The in vitro models of (human) adipose tissue-derived stem cells (hADMSCs), (embryonic) fibroblasts, or immortalized cell lines (e.g., 3T3-L1, 3T3-F442A, etc.) (Ruiz-Ojeda et al. 2016) are useful tools in understanding transcriptional control over adipogenesis. The differentiation protocol usually involves a complete stop of proliferation by growing cells at confluency, followed by the induction of differentiation by a cocktail of hormones including insulin, a synthetic glucocorticoid, dexamethasone, and 3-isobutyl-1-methylxanthine (IBMX), a phosphodiesterase inhibitor. After the induction of differentiation, cells undergo commitment and committed cells undergo a few rounds of cellular division, called mitotic clonal expansion (Fig. 2). It is not known whether clonal expansion also characterizes the in vivo differentiation of adipocytes. After clonal expansion, cells begin accumulating lipids in lipid droplets (in vitro differentiated adipocytes are multilocular), termed terminal differentiation (Fig. 2; Ruiz-Ojeda et al. 2016; Mota de Sa et al. 2017).

Concerted action of a large set of transcription factors is needed to guide adipogenic differentiation ( (Fig. 2; Mota de Sa et al. 2017). Adipogenic transcription factors interacting with PARPs are listed in Table 3. Classically, clonal expansion of white adipocytes was shown to be mediated by the self-amplifying activation of C/EBPδ and C/EBPβ that subsequently induces the expression of C/EBPα and, finally, the expression of peroxisome proliferator activated receptor (PPAR) γ1 and PPARγ2 expression (Fajas et al. 1998).

**Table 3.**
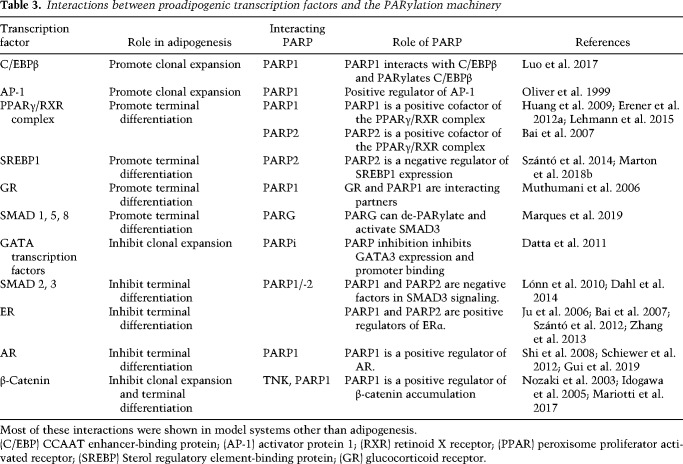
Interactions between proadipogenic transcription factors and the PARylation machinery

PPARγ1 and PPARγ2 belong to the family of nuclear receptors and are crucial in driving adipogenesis and adipocyte function through supporting the expression of major adipogenic genes (Fajas et al. 1998). PPARγ-dependent genes include lipoprotein lipase (LPL), fatty acid transporters (CD36 and aP2), TG storage proteins (e.g., perilipin), and adipokines (e.g., leptin, adiponectin) (Auwerx et al. 2003). While PPARγ1 is expressed ubiquitously, PPARγ2 expression is restricted to adipocytes and macrophages (Fajas et al. 1997; Nagy et al. 1998). Both PPARγ isoforms are lipid activated, suggesting an intricate modulation of PPARγ activity by lipid species (Nagy et al. 1998). The ligand-mediated activation of PPARγ involves the exchange of repressor cofactors (e.g., NCoR-1) to coactivator factors (e.g., p300) that facilitate chromatin relaxation and the initiation of transcription (Gelman et al. 1999; Coste et al. 2008).

The induction of the expression of PPARγ isoforms is a common denominator of beige and brown adipogenesis, similar to white adipogenesis. Mitochondrial biogenesis is a key factor for the differentiation of beige and brown adipocytes. The concerted action of the energy stress sensor web is vital for the induction of mitochondrial biogenesis, including the activation of AMPK or SIRT1 (Qiang et al. 2012; Shan et al. 2013; Wang et al. 2015; Abdul-Rahman et al. 2016; Nagy et al. 2019).

## The role of PARP enzymes in adipogenesis

The first observation that PARPs modulate adipogenesis came in 1995 by Smulson et al. (1995) using the 3T3-L1 model system and 3AB, a rather unspecific PARP inhibitor. This study showed that pharmacological PARP inhibition hampers 3T3-L1 differentiation (Smulson et al. 1995). Indeed, PARPs play a role in the regulation of adipogenesis and adipose tissue function. Since this first observation, much data has emerged along with numerous controversial issues.

### Early commitment and clonal expansion

PARP1, PARP2, and PARP7 have pivotal roles in decision making between retaining stem cell properties and differentiation in nonadipogenic models (Yélamos et al. 2006; Farrés et al. 2013, 2015; Nozaki et al. 2013; Roper et al. 2014; Vida et al. 2016). Therefore, PARPs may be crucial in the early commitment of cells toward preadipocytes and adipose lineages (Fig. 1). To date, no studies have been published concerning the role of PARPs in commitment to adipocyte lineages in an in vivo setting (e.g., as in Sanchez-Gurmaches and Guertin 2014b). However, PARP1 has a crucial role in preadipocyte commitment to white adipocyte differentiation in in vitro systems (Luo et al. 2017; Ryu et al. 2018).

In the in vitro differentiation of 3T3-L1 preadipocytes, a characteristic PARylation pattern was detected (Luo et al. 2017). In confluency (growth arrest), PARP1 auto-PARylation dominates cells, after which the PARylation signal is low in the clonal expansion phase and boosts again in terminal differentiation (Luo et al. 2017). In terminal differentiation, PARP1 auto-PARylation returns, nevertheless, lower molecular weight PARylation signals are also detected (Luo et al. 2017).

As noted in the previous chapter, the clonal expansion phase is dominated by the self-intensifying loop between C/EBPβ and C/EBPδ. This loop is vital for the subsequent transcription of C/EBPα and PPARγ transcription factors that then transcribe the “executors” of lipogenesis. PARP1 can PARylate C/EBPβ on K133, E135, and E139 residues, resulting in decreased binding of C/EBPβ to the promoters of *C/EBPα* or *PPARγ2*. Hence, genetic or pharmacological inactivation of PARP1 supports adipocyte differentiation (Luo et al. 2017). The deletion of these PARylation sites enhance C/EBPβ binding to target promoters and renders C/EBPβ resistant to PARP inhibitors. These findings provide a physiological explanation for reduced PARylation during the clonal expansion phase.

Another mechanism for the regulation of PARP1 activity and clonal expansion is the compartment-specific NAD^+^ biosynthesis through NMNAT enzymes. Ryu et al. (2018) showed that blocking nuclear NMNAT-1 induces adipocyte differentiation through limiting nuclear NAD^+^ for PARP1. In other words, PARP1 activation and fueling PARP1 activation by NMNAT-1 can keep preadipocytes undifferentiated. The cytosolic NMNAT-2 is induced early in adipocyte differentiation (4 h after induction) and shifts nuclear NAD^+^ biosynthesis to the cytosol to support glycolysis (Ryu et al. 2018). As a “side effect,” nuclear PARylation is reduced, supporting white adipocyte differentiation (Ryu et al. 2018).

### Adipocyte terminal differentiation

Adipocyte terminal differentiation in in vitro models is characterized by increasing C/EBPα and PPARγ protein expression and lipid accumulation. This phase of terminal differentiation is associated with the accumulation of PARP1 and PAR formation (Erener et al. 2012a; Luo et al. 2017). In the studies of Erener et al. (2012a,b), pharmacological and genetic PARP inhibition blocked the differentiation of 3T3-L1 cells. When PARP1 was blocked in the course of 3T3-L1 differentiation, a major reduction in the expression of C/EBPα and PPARγ2 and a set of PPARγ-dependent transcripts was observed, in stark contrast to the previously discussed studies (Luo et al. 2017; Ryu et al. 2018).

Lower adipocyte differentiation was linked to a slower resolution of transcription-coupled topoisomerase II-inflicted double strand breaks and the consequent slower initiation of RNA polymerase II-mediated transcription in the absence of PARP activity (Pavri et al. 2005; Erener et al. 2012a; Lehmann et al. 2015). Furthermore, PARP inhibition supported the binding of NCoR-1 (an inhibitory cofactor of PPARγ), while decreasing the binding of p300 (an activating cofactor of PPARγ) (Lehmann et al. 2015). In a cardiomyocyte model, pharmacological, and genetic PARP1 inhibition led to increased PPARγ activity (Huang et al. 2009), in contrast to the observations detailed above.

There is apparent contradiction between the results showing that PARP1 and NAD^+^ biosynthesis during the commitment phase blocks (Luo et al. 2017; Ryu et al. 2018), while during terminal differentiation PARP1 supports adipocyte differentiation (Erener et al. 2012a,b; Lehmann et al. 2015). To date, no explanation is given to the discrepancies that is backed by experimental proof. Nevertheless, the visibly contradictory results may be both true. The contradictory reports do observe PARP auto-PARylation in confluent and in terminally differentiated cells (Erener et al. 2012a; Luo et al. 2017) suggesting that similar processes may take place in all cases; however, the dependence of the cells on early commitment may be different. In our hands different clones of the 3T3-L1 cells have different behavior in differentiation and response to PARP inhibitors (unpublished data).

The genetic silencing of PARP2 led to lipodystophy in chow diet-fed mice, which was mirrored when primary fibroblasts were differentiated to mature adipocytes (Bai et al. 2007). Decreased adipocytic differentiation was a result of blunted PPARγ activation. PARP2 binds to PPARγ-mediated promoters (e.g., *aP2*) and supports mRNA transcription. Reduced expression of the PPARγ-dependent genes in the PARP2 knockout mice points toward hampered PPARγ activation in the absence of PARP2 (Bai et al. 2007).

In the above-mentioned studies (Bai et al. 2007; Huang et al. 2009; Erener et al. 2012a,eb; Lehmann et al. 2015; Luo et al. 2017; Ryu et al. 2018), PARP inhibition or the genetic deletion of *PARP1* or *PARP2* modulated genes involved in fatty acid uptake (lipoprotein lipase [*LPL*], fatty acid binding protein 4 [*FABP4*, *aP2*], and *CD36*), lipid storage (*perilipin*), fatty acid biosynthesis (fatty acid synthase [*FAS*]), and adipokines (*leptin*, *adiponectin*, and *resistin*) in white adipocyte differentiation models. The deletion of *tankyrase-1* induced leptin and adiponectin expression and secretion from white adipose tissue (Yeh et al. 2009). These genes are PPARγ-dependent and encompass all processes needed for triglyceride uptake and storage. To date, no studies have reported fatty acid release disorders in relation to the modulation of PARP1 or PARP2 activity (Bai et al. 2007; Erener et al. 2012b).

### Switch between white, brown, or beige adipogenesis

PARPs may have a role in selecting between the differentiation to white, brown, and beige adipocytes. PARP1 and PARP2 were shown to modulate skeletal muscle myoblast differentiation and health (Butler and Ordahl 1999; Vyas et al. 2001; Hu et al. 2013; Chacon-Cabrera et al. 2015). Therefore, it is also likely that PARPs can influence white/brown/beige diversion. This hypothesis is further supported by the widespread interactions between energy stress sensors, mitochondrial biogenesis regulators, and PARPs (Bai et al. 2015).

The deletion of *PARP1* or *PARP2*, as well as the pharmacological inhibition of PARP, supports mitochondrial biogenesis (Virag et al. 1998a; Bai et al. 2011a,b,^2015^; Szanto et al. 2011; Mohamed et al. 2014) via the preservation of cellular NAD^+^ pools and the subsequent activation of the SIRT1–PGC1α axis (Cantó et al. 2013; Bai et al. 2015). In agreement with this, Nagy et al. (2019) found that in vitro treatment of hADMSC cells, differentiated to white adipocytes, with olaparib induced browning of the cells, marked by mitochondrial biogenesis and UCP1 induction. In the olaparib-treated cells, beige cell markers were not induced, suggesting browning induced transdifferentiation to brown adipocytes. In good agreement with that observation, in *PARP1* knockout mice, we detected more active brown adipose tissue (lower lipid deposition, induction of UCPs, increased fatty acid oxidation, and higher mitochondrial content), increased energy expenditure, and improved capacity to withstand cold exposure (Bai et al. 2011b). We detected increased cellular NAD^+^ content and SIRT1 activity in both models (Bai et al. 2011b; Nagy et al. 2019). Interestingly, the brown adipose tissue of the *PARP2* knockout mice was not more active (Bai et al. 2011a). To date, no thorough studies were performed to assess the contribution of PARPs to beige and brown adipocyte differentiation. These findings are in agreement with the observations that better NAD^+^ availability (Yamaguchi et al. 2019) or SIRT1 activation supports brown and beige differentiation (Qiang et al. 2012; Khanh et al. 2018).

### Lipid accumulation, obesity, insulin sensitivity

A role for PARP enzymes in obesity has been reported. In a study of monozygotic twins, higher PARP activity was found in the subcutaneous white adipose tissue of the heavier cotwin (Jukarainen et al. 2016). Furthermore, in weight loss adipocytic PARP activity is reduced, while SIRT1 activity is up-regulated (Rappou et al. 2016). In murine studies, PARP1, PARP2, and tankyrase-1 were shown to be involved in modulating energy balance and obesity. Similar to the ambiguity in the role of PARP1 in adipocyte differentiation, the studies on the organismal role of PARP1 in obesity and its consequences are also contradictory. In our studies, *PARP1* knockout mice were leaner when kept on chow diet that was accentuated on high-fat feeding (Bai et al. 2011b). This study was backed by a study from another laboratory. PARP1 knockout mice had lower body weight and white adipose tissue mass when on a high-fat diet (Erener et al. 2012b). Furthermore, treatment of mice with an orally administered PARP inhibitor, MRLB-45696, (PARP1 is responsible for 80%–85% of total cellular PARP activity) (Schreiber et al. 2002; Szanto et al. 2011) prevented weight gain on a high-fat diet (Lehmann et al. 2015). In contrast to these studies, a report by Devalaraja-Narashimha and Padanilam (2010) reported a complete opposite phenotype; the *PARP1* knockout mice became seriously obese as compared with their wild-type counter partners upon high-fat feeding. In all studies, a hypercaloric high-fat diet was used.

Obesity is a complex pathology and cannot be solely attributed to the dysfunction of white adipocytes; a complex deregulation of organismal energy homeostasis is involved (Rosen and Spiegelman 2014). In the above-mentioned studies that reported a lean phenotype, an energy expenditure phenotype was described due to mitochondrial biogenesis in the brown adipose tissue and the skeletal muscle, attributed mainly to the activation of the NAD^+^–SIRT1 axis (Bai et al. 2011b; Pirinen et al. 2014; Lehmann et al. 2015). The improved metabolic fitness yielded improved glucose tolerance and insulin sensitivity, with skeletal muscle being responsible for glucose clearance both in chow-fed and high-fat-fed mice (Bai et al. 2011b). In the monozygotic twin study, the activation of the NAD^+^–SIRT1 axis and the consequently lower PARP activity was associated with a leaner, metabolically healthier phenotype (Jukarainen et al. 2016). The contradictory study (Devalaraja-Narashimha and Padanilam 2010) reported an opposing rearrangement of energy homeostasis characterized by lower oxygen consumption, energy deliberation, worsened glucose clearance, and insulin resistance.

These are again opposing results without good experimental explanation. A root cause for the disagreement between the studies could be that these studies were conducted on two different knockout *PARP1* mouse strains. One of the strains was generated by Wang et al. (1995) and deposited at Jackson Laboratories; the other strain was generated in the laboratory of de Murcia et al. (1997). The mice generated by Wang et al. (1995) were on an SV129 background, while the mice generated by de Murcia et al. (1997) were on a C57/Bl6J background. The metabolic behavior of the two different backgrounds is profoundly different (Andrikopoulos et al. 2005; Berglund et al. 2008) and might be the explanation for the differing results. A solution for these issues could be the use of a transgenic *PARP1loxP* mouse strain that will bypass developmental issues and enable the study of interorgan interactions (JAX 2019).

Induction of mitochondrial biogenesis by enhancing the NAD^+^–SIRT1 axis in the skeletal muscle after the genetic deletion of *PARP2* brought about a lean phenotype (Bai et al. 2011a; Mohamed et al. 2014). Interestingly, the brown adipose tissue of the *PARP2* knockout mice was not involved in the energy expenditure phenotype, in contrast to the *PARP1* knockout mice (Bai et al. 2011a,b). In chow-fed mice, the deletion of *PARP2* improved insulin sensitivity and glucose clearance. While on a high-fat diet, the ablation of *PARP2* improved insulin sensitivity, but insulin secretion and glucose clearance were blunted due to inhibition of compensatory β-cell proliferation (Bai et al. 2011a).

Tankyrase expression is among the highest in the white adipose tissue and the brain (Yeh et al. 2009). White adipose tissue and energy homeostasis changes were observed in *tankyrase* knockout mice (Yeh et al. 2007, 2009). Interestingly, tankyrase expression may also affect brown adipose tissue (Yeh et al. 2009). However, the involvement of tankyrase in brown adipose tissue function was not investigated yet. Tankyrase knockdown was shown to impair Glut4 translocation and hence insulin-stimulated glucose uptake, resulting in down-regulation of glucose metabolism in differentiated 3T3-L1 adipocytes (Yeh et al. 2007). These effects were dependent on tankyrase activity (Yeh et al. 2007). In *tankyrase* knockout mice, the relative mass of the epididymal white adipose tissue decreased in parallel to enhanced energy expenditure marked by increased oxygen consumption (Yeh et al. 2009).

## Future directions

PARP enzymes and PARP inhibition interfere with adipose tissue biology at multiple points. There are obesity-associated processes (e.g., inflammation) that are also PARP regulated, but their interplay had not been assessed. We give a brief overview of these processes below.

Inflammation plays diverse roles in obesity and adipose tissue homeostasis. Obesity is associated with inflammation and fibrosis of the adipose tissue (Reilly and Saltiel 2017). Preventing adipose tissue inflammation is a key step toward the “metabolically healthy” obese phenotype (Vishvanath and Gupta 2019). Furthermore, inflammatory signaling seems to be a player in diverting toward the beige lineage (Sun et al. 2018). PARP enzymes are involved in the regulation of inflammation; usually, the absence of PARP1 or PARP2 or pharmacological PARP inhibition is anti-inflammatory (Fehr et al. 2020), except for Th17-mediated processes (Kiss et al. 2019). Furthermore, increases in SIRT1 activity, which can be elicited by PARP inhibition, can suppress adipose tissue inflammation, and hence support its function (Gillum et al. 2011; Chalkiadaki and Guarente 2012). Importantly, there is evidence that the results of murine PARP inhibitor studies are likely translatable to humans (Morrow et al. 2009). PARP1 and PARP inhibition regulate IL6 (Lehmann et al. 2015), IL12m, IL13ra, SAA3, pu1, and MPEG1 (Erener et al. 2012b) expression. In the adipose tissue of *PARP2* knockout mice, signs of inflammation were detected, including F4/80 positive cells and dilated capillaries, that were absent in their wild-type counter partners (Bai et al. 2007). Whether inflammatory processes are the cause or consequence of the distortion of adipose tissue function is unknown.

Recent studies showed that the loss of microbiome diversity hampers adipose tissue browning (Suárez-Zamorano et al. 2015; Li et al. 2019). Intriguingly, the genetic deletion of PARP1 enhances the diversity of the gut microbiome (Larmonier et al. 2016; Vida et al. 2018), suggesting a possible link between PARP1 and adipose tissue browning. Disruption of circadian entrainment of feeding can also contribute to obesity (Hatori et al. 2012; Zarrinpar et al. 2016; Chaix et al. 2019) and, as noted earlier, the disruption of PARP1 leads to changes in the diurnal cycle of feeding and metabolism (Asher et al. 2010; Bai et al. 2011b). PARP activation can be a go/no-go signal in cell death (Virág et al. 1998b; Fatokun et al. 2014; Dawson and Dawson 2017) and PARPs regulate cellular proliferation (Bai 2015), two vital steps to adipocyte differentiation and selection between beige, brown, or white lineages. Similarly, PARP1 and PARP10 were implicated in the regulation of autophagy and mitophagy (Muñoz-Gámez et al. 2009; Kleine et al. 2012), processes that shape adipocyte differentiation (Kim and Lee 2014). PARPs affect nuclear structure and the epigenetic code (Wacker et al. 2007; Krishnakumar et al. 2008; Hottiger 2015; Zhao et al. 2015). PARP1 deficiency was shown to modulate H3K9me3 and H3K4me3 methylation during adipogenic differentiation (Erener et al. 2012a). Nevertheless, large scale studies are missing. There are genes reported to be PARP-mediated (e.g., *MDH1*) (Hopp et al. 2019) that regulate adipocyte differentiation. Again, the involvement of these genes in adipogenesis in the context of PARylation had not been assessed.

All adipose tissue depots are characterized by secretion of bioactive compounds such as peptide hormones (adipokines), bioactive lipids (lipokines), and RNA molecules with local (paracrine) and systemic (endocrine) effects on multiple metabolic tissues and the cardiovascular system. These bioactive compounds are synthesized and secreted as a function of the energy status of adipose tissues, which in turn regulates appetite, thermogenesis, glucose, and lipid metabolism (Scheja and Heeren 2019). The role of PARPs had not been studied in this direction. Along the same lines, large-scale endocrine studies are also missing.

The role of PARPs in adipogenesis and metabolism will clearly have practical applications not only in the strict sense of metabolism and metabolic diseases, but also from the perspective of cancer and cancer cachexia (Chacon-Cabrera et al. 2015, 2017; Barreiro and Gea 2018; Doles et al. 2018). These outstanding issues warrant further studies in the future.

## References

[GAD334284SZAC1] Abdul-Rahman O, Kristóf E, Doan-Xuan QM, Vida A, Nagy L, Horváth A, Simon J, Maros T, Szentkirályi I, Palotás L, 2016 AMP-activated kinase (AMPK) activation by AICAR in human white adipocytes derived from pericardial white adipose tissue stem cells induces a partial beige-like phenotype. PLoS One 11: e0157644 10.1371/journal.pone.0157644 27322180 PMC4913939

[GAD334284SZAC2] Abplanalp J, Hopp AK, Hottiger MO. 2018 Mono-ADP-ribosylhydrolase assays. Methods Mol Biol 1813: 205–213. 10.1007/978-1-4939-8588-3_13 30097869

[GAD334284SZAC3] Ambrose HE, Willimott S, Beswick RW, Dantzer F, de Murcia JM, Yelamos J, Wagner SD. 2009 Poly(ADP-ribose) polymerase-1 (Parp-1)-deficient mice demonstrate abnormal antibody responses. Immunology 127: 178–186. 10.1111/j.1365-2567.2008.02921.x 18778284 PMC2691783

[GAD334284SZAC4] Amé JC, Rolli V, Schreiber V, Niedergang C, Apiou F, Decker P, Muller S, Höger T, Menissier-de Murcia J, de Murcia G. 1999 PARP-2, A novel mammalian DNA damage-dependent poly(ADP-ribose) polymerase. J Biol Chem 274: 17860–17868. 10.1074/jbc.274.25.17860 10364231

[GAD334284SZAC5] Amé JC, Spenlehauer C, de Murcia G. 2004 The PARP superfamily. Bioessays 26: 882–893. 10.1002/bies.20085 15273990

[GAD334284SZAC6] Amin O, Beauchamp MC, Nader PA, Laskov I, Iqbal S, Philip CA, Yasmeen A, Gotlieb WH. 2015 Suppression of homologous recombination by insulin-like growth factor-1 inhibition sensitizes cancer cells to PARP inhibitors. BMC Cancer 15: 817 10.1186/s12885-015-1803-y 26510816 PMC4625613

[GAD334284SZAC7] Andrabi SA, Umanah GK, Chang C, Stevens DA, Karuppagounder SS, Gagne JP, Poirier GG, Dawson VL, Dawson TM. 2014 Poly(ADP-ribose) polymerase-dependent energy depletion occurs through inhibition of glycolysis. Proc Natl Acad Sci 111: 10209–10214. 10.1073/pnas.1405158111 24987120 PMC4104885

[GAD334284SZAC8] Andrikopoulos S, Massa CM, Aston-Mourney K, Funkat A, Fam BC, Hull RL, Kahn SE, Proietto J. 2005 Differential effect of inbred mouse strain (C57BL/6, DBA/2, 129T2) on insulin secretory function in response to a high fat diet. J Endocrinol 187: 45–53. 10.1677/joe.1.06333 16214940

[GAD334284SZAC9] Asher G, Reinke H, Altmeyer M, Gutierrez-Arcelus M, Hottiger MO, Schibler U. 2010 Poly(ADP-ribose) polymerase 1 participates in the phase entrainment of circadian clocks to feeding. Cell 142: 943–953. 10.1016/j.cell.2010.08.016 20832105

[GAD334284SZAC10] Auwerx J, Cock TA, Knouff C. 2003 PPAR-γ: a thrifty transcription factor. Nucl Recept Signal 1: e006. 16604178 10.1621/nrs.01006 PMC1402226

[GAD334284SZAC11] Bai P. 2015 Biology of poly(ADP-ribose) polymerases: the factotums of cell maintenance. Mol Cell 58: 947–958. 10.1016/j.molcel.2015.01.034 26091343

[GAD334284SZAC12] Bai P, Cantó C. 2012 The role of PARP-1 and PARP-2 enzymes in metabolic regulation and disease. Cell Metab 16: 290–295. 10.1016/j.cmet.2012.06.016 22921416

[GAD334284SZAC13] Bai P, Csóka B. 2015 New route for the activation of poly(ADP-ribose) polymerase-1: a passage that links poly(ADP-ribose) polymerase-1 to lipotoxicity? Biochem J 469: e9–e11. 10.1042/BJ20150598 26171833

[GAD334284SZAC14] Bai P, Houten SM, Huber A, Schreiber V, Watanabe M, Kiss B, de Murcia G, Auwerx J, Menissier-de Murcia J. 2007 Poly(ADP-ribose) polymerase-2 controls adipocyte differentiation and adipose tissue function through the regulation of the activity of the retinoid X receptor/peroxisome proliferator-activated receptor-γ heterodimer. J Biol Chem 282: 37738–37746. 10.1074/jbc.M701021200 17951580

[GAD334284SZAC15] Bai P, Canto C, Brunyánszki A, Huber A, Szántó M, Cen Y, Yamamoto H, Houten SM, Kiss B, Oudart H, 2011a PARP-2 regulates SIRT1 expression and whole-body energy expenditure. Cell Metab 13: 450–460. 10.1016/j.cmet.2011.03.013 21459329 PMC3108571

[GAD334284SZAC16] Bai P, Cantó C, Oudart H, Brunyánszki A, Cen Y, Thomas C, Yamamoto H, Huber A, Kiss B, Houtkooper RH, 2011b PARP-1 inhibition increases mitochondrial metabolism through SIRT1 activation. Cell Metab 13: 461–468. 10.1016/j.cmet.2011.03.004 21459330 PMC3086520

[GAD334284SZAC17] Bai P, Nagy L, Fodor T, Liaudet L, Pacher P. 2015 Poly(ADP-ribose) polymerases as modulators of mitochondrial activity. Trends Endocrinol Metab 26: 75–83. 10.1016/j.tem.2014.11.003 25497347

[GAD334284SZAC18] Barkauskaite E, Jankevicius G, Ladurner AG, Ahel I, Timinszky G. 2013 The recognition and removal of cellular poly(ADP-ribose) signals. FEBS J 280: 3491–3507. 10.1111/febs.12358 23711178

[GAD334284SZAC19] Barreiro E, Gea J. 2018 PARP-1 and PARP-2 activity in cancer-induced cachexia: potential therapeutic implications. Biol Chem 399: 179–186. 10.1515/hsz-2017-0158 29016348

[GAD334284SZAC20] Berger F, Lau C, Ziegler M. 2007 Regulation of poly(ADP-ribose) polymerase 1 activity by the phosphorylation state of the nuclear NAD biosynthetic enzyme NMN adenylyl transferase 1. Proc Natl Acad Sci 104: 3765–3770. 10.1073/pnas.0609211104 17360427 PMC1820658

[GAD334284SZAC21] Berglund ED, Li CY, Poffenberger G, Ayala JE, Fueger PT, Willis SE, Jewell MM, Powers AC, Wasserman DH. 2008 Glucose metabolism in vivo in four commonly used inbred mouse strains. Diabetes 57: 1790–1799. 10.2337/db07-1615 18398139 PMC2453626

[GAD334284SZAC22] Bertholet AM, Kazak L, Chouchani ET, Bogaczynska MG, Paranjpe I, Wainwright GL, Bétourne A, Kajimura S, Spiegelman BM, Kirichok Y. 2017 Mitochondrial patch clamp of beige adipocytes reveals UCP1-positive and UCP1-negative cells both exhibiting futile creatine cycling. Cell Metab 25: 811–822.e4. 10.1016/j.cmet.2017.03.002 28380374 PMC5448977

[GAD334284SZAC23] Bianchi AR, Ferreri C, Ruggiero S, Deplano S, Sunda V, Galloro G, Formisano C, Mennella MR. 2016 Automodification of PARP and fatty acid-based membrane lipidome as a promising integrated biomarker panel in molecular medicine. Biomark Med 10: 229–242. 10.2217/bmm.16.3 26860237

[GAD334284SZAC24] Billon N, Iannarelli P, Monteiro MC, Glavieux-Pardanaud C, Richardson WD, Kessaris N, Dani C, Dupin E. 2007 The generation of adipocytes by the neural crest. Development 134: 2283–2292. 10.1242/dev.002642 17507398 PMC6334830

[GAD334284SZAC25] Bindesboll C, Tan S, Bott D, Cho T, Tamblyn L, MacPherson L, Gronning-Wang L, Nebb HI, Matthews J. 2016 TCDD-inducible poly-ADP-ribose polymerase (TIPARP/PARP7) mono-ADP-ribosylates and co-activates liver X receptors. Biochem J 473: 899–910. 10.1042/BJ20151077 26814197

[GAD334284SZAC26] Bodnár E, Bakondi E, Kovács K, Hegedűs C, Lakatos P, Robaszkiewicz A, Regdon Z, Virág L, Szabó E. 2018 Redox profiling reveals clear differences between molecular patterns of wound fluids from acute and chronic wounds. Oxid Med Cell Longev 2018: 1–12. 10.1155/2018/5286785 PMC6276414 30581533

[GAD334284SZAC27] Burkart V, Blaeser K, Kolb H. 1999 Potent β-cell protection in vitro by an isoquinolinone-derived PARP inhibitor. Horm Metab Res 31: 641–644. 10.1055/s-2007-978813 10668915

[GAD334284SZAC28] Burkle A, Virag L. 2013 Poly(ADP-ribose): PARadigms and PARadoxes. Mol Aspects Med 2: 00157–00154. 10.1016/j.mam.2012.12.010 23290998

[GAD334284SZAC29] Butler AJ, Ordahl CP. 1999 Poly(ADP-ribose) polymerase binds with transcription enhancer factor 1 to MCAT1 elements to regulate muscle-specific transcription. Mol Cell Biol 19: 296–306. 9858553 10.1128/mcb.19.1.296 PMC83887

[GAD334284SZAC30] Cannon B, Nedergaard J. 2004 Brown adipose tissue: function and physiological significance. Physiol Rev 84: 277–359. 10.1152/physrev.00015.2003 14715917

[GAD334284SZAC31] Cantó C, Sauve A, Bai P. 2013 Crosstalk between poly(ADP-ribose) polymerase and sirtuin enzymes. Mol Asp Med 34: 1168–1201. 10.1016/j.mam.2013.01.004 PMC3676863 23357756

[GAD334284SZAC32] Cedernaes J, Waldeck N, Bass J. 2019 Neurogenetic basis for circadian regulation of metabolism by the hypothalamus. Genes Dev 33: 1136–1158. 10.1101/gad.328633.119 31481537 PMC6719618

[GAD334284SZAC33] Chacon-Cabrera A, Fermoselle C, Salmela I, Yelamos J, Barreiro E. 2015 MicroRNA expression and protein acetylation pattern in respiratory and limb muscles of Parp-1^−/−^ and Parp-2^−/−^ mice with lung cancer cachexia. Biochim Biophys Acta 1850: 2530–2543. 10.1016/j.bbagen.2015.09.020 26432600

[GAD334284SZAC34] Chacon-Cabrera A, Mateu-Jimenez M, Langohr K, Fermoselle C, García-Arumi E, Andreu AL, Yelamos J, Barreiro E. 2017 Role of PARP activity in lung cancer-induced cachexia: effects on muscle oxidative stress, proteolysis, anabolic markers, and phenotype. J Cell Physiol 232: 3744–3761. 10.1002/jcp.25851 28177129

[GAD334284SZAC35] Chaix A, Lin T, Le HD, Chang MW, Panda S. 2019 Time-restricted feeding prevents obesity and metabolic syndrome in mice lacking a circadian clock. Cell Metab 29: 303–319.e4. 10.1016/j.cmet.2018.08.004 30174302 PMC7751278

[GAD334284SZAC36] Chalkiadaki A, Guarente L. 2012 High-fat diet triggers inflammation-induced cleavage of SIRT1 in adipose tissue to promote metabolic dysfunction. Cell Metab 16: 180–188. 10.1016/j.cmet.2012.07.003 22883230 PMC3539750

[GAD334284SZAC37] Chambon P, Weill JD, Mandel P. 1963 Nicotinamide mononucleotide activation of new DNA-dependent polyadenylic acid synthesizing nuclear enzyme. Biochem Biophys Res Commun 11: 39–43. 14019961 10.1016/0006-291x(63)90024-x

[GAD334284SZAC38] Chapman JD, Gagné JP, Poirier GG, Goodlett DR. 2013 Mapping PARP-1 auto-ADP-ribosylation sites by liquid chromatography-tandem mass spectrometry. J Proteome Res 12: 1868–1880. 10.1021/pr301219h 23438649

[GAD334284SZAC39] Chen ST, Lin CC, Liu YS, Lin C, Hung PT, Jao CW, Lin PH. 2013 Airborne particulate collected from central Taiwan induces DNA strand breaks, Poly(ADP-ribose) polymerase-1 activation, and estrogen-disrupting activity in human breast carcinoma cell lines. J Environ Sci Health A Tox Hazard Subst Environ Eng 48: 173–181. 10.1080/10934529.2012.717809 23043339

[GAD334284SZAC40] Chen Y, Bang S, Park S, Shi H, Kim SF. 2015 Acyl-CoA-binding domain containing 3 modulates NAD^+^ metabolism through activating poly(ADP-ribose) polymerase 1. Biochem J 469: 189–198. 10.1042/BJ20141487 25940138

[GAD334284SZAC41] Chiarugi A, Dolle C, Felici R, Ziegler M. 2012 The NAD metabolome - a key determinant of cancer cell biology. Nat Rev Cancer 2012: 13. 10.1038/nrc3340 23018234

[GAD334284SZAC42] Cho SH, Ahn AK, Bhargava P, Lee CH, Eischen CM, McGuinness O, Boothby M. 2011 Glycolytic rate and lymphomagenesis depend on PARP14, an ADP ribosyltransferase of the B aggressive lymphoma (BAL) family. Proc Natl Acad Sci 108: 15972–15977. 10.1073/pnas.1017082108 21911376 PMC3179111

[GAD334284SZAC43] Choi Y, Abdelmegeed MA, Song BJ. 2017 Diet high in fructose promotes liver steatosis and hepatocyte apoptosis in C57BL/6J female mice: role of disturbed lipid homeostasis and increased oxidative stress. Food Chem Toxicol 103: 111–121. 10.1016/j.fct.2017.02.039 28257781 PMC5499254

[GAD334284SZAC44] Claussnitzer M, Dankel SN, Kim KH, Quon G, Meuleman W, Haugen C, Glunk V, Sousa IS, Beaudry JL, Puviindran V, 2015 *FTO* obesity variant circuitry and adipocyte browning in humans. N Engl J Med 373: 895–907. 10.1056/NEJMoa1502214 26287746 PMC4959911

[GAD334284SZAC45] Cohen MS. 2020 Interplay between compartmentalized NAD^+^ synthesis and consumption: a focus on the PARP family. Genes Dev (this issue) 10.1101/gad.335109.119 PMC7050480 32029457

[GAD334284SZAC46] Coste A, Louet JF, Lagouge M, Lerin C, Antal MC, Meziane H, Schoonjans K, Puigserver P, O'Malley BW, Auwerx J. 2008 The genetic ablation of SRC-3 protects against obesity and improves insulin sensitivity by reducing the acetylation of PGC-1α. Proc Natl Acad Sci 105: 17187–17192. 10.1073/pnas.0808207105 18957541 PMC2579399

[GAD334284SZAC47] Curtin N, Szabo C. 2013 Therapeutic applications of PARP inhibitors: anticancer therapy and beyond. Mol Aspects Med 6: 1043–1258. 10.1016/j.mam.2013.01.006 PMC3657315 23370117

[GAD334284SZAC48] Dahl M, Maturi V, Lönn P, Papoutsoglou P, Zieba A, Vanlandewijck M, van der Heide LP, Watanabe Y, Söderberg O, Hottiger MO, 2014 Fine-tuning of Smad protein function by poly(ADP-ribose) polymerases and poly(ADP-ribose) glycohydrolase during transforming growth factor β signaling. PLoS One 9: e103651 10.1371/journal.pone.0103651 25133494 PMC4136792

[GAD334284SZAC49] Dantzer F, Santoro R. 2013 The expanding role of PARPs in the establishment and maintenance of heterochromatin. FEBS J 280: 3508–3518. 10.1111/febs.12368 23731385

[GAD334284SZAC50] Datta R, Naura AS, Zerfaoui M, Errami Y, Oumouna M, Kim H, Ju J, Ronchi VP, Haas AL, Boulares AH. 2011 PARP-1 deficiency blocks IL-5 expression through calpain-dependent degradation of STAT-6 in a murine asthma model. Allergy 66: 853–861. 10.1111/j.1398-9995.2011.02549.x 21276008 PMC3150522

[GAD334284SZAC51] Dawson TM, Dawson VL. 2017 Mitochondrial mechanisms of neuronal cell death: potential therapeutics. Annu Rev Pharmacol Toxicol 57: 437–454. 10.1146/annurev-pharmtox-010716-105001 28061689 PMC11323062

[GAD334284SZAC52] de Murcia JM, Niedergang C, Trucco C, Ricoul M, Dutrillaux B, Mark M, Oliver FJ, Masson M, Dierich A, LeMeur M, 1997 Requirement of poly(ADP-ribose) polymerase in recovery from DNA damage in mice and in cells. Proc Natl Acad Sci 94: 7303–7307. 10.1073/pnas.94.14.7303 9207086 PMC23816

[GAD334284SZAC53] Devalaraja-Narashimha K, Padanilam BJ. 2009 PARP-1 inhibits glycolysis in ischemic kidneys. J Am Soc Nephrol 20: 95–103. 10.1681/ASN.2008030325 19056868 PMC2615730

[GAD334284SZAC54] Devalaraja-Narashimha K, Padanilam BJ. 2010 PARP1 deficiency exacerbates diet-induced obesity in mice. J Endocrinol 205: 243–252. 10.1677/JOE-09-0402 20338998

[GAD334284SZAC55] De Vos M, Schreiber V, Dantzer F. 2012 The diverse roles and clinical relevance of PARPs in DNA damage repair: current state of the art. Biochem Pharmacol 84: 137–146. 10.1016/j.bcp.2012.03.018 22469522

[GAD334284SZAC56] Diestel A, Aktas O, Hackel D, Häke I, Meier S, Raine CS, Nitsch R, Zipp F, Ullrich O. 2003 Activation of microglial poly(ADP-ribose)-polymerase-1 by cholesterol breakdown products during neuroinflammation: a link between demyelination and neuronal damage. J Exp Med 198: 1729–1740. 10.1084/jem.20030975 14657223 PMC2194134

[GAD334284SZAC57] Doles JD, Hogan KA, O'Connor J, Wahner Hendrickson AE, Huston O, Jatoi A. 2018 Does the Poly (ADP-Ribose) polymerase inhibitor veliparib merit further study for cancer-associated weight loss? Observations and conclusions from 60 prospectively treated patients. J Palliat Med 21: 1334–1338. 10.1089/jpm.2018.0023 29792535 PMC6154444

[GAD334284SZAC58] Du X, Matsumura T, Edelstein D, Rossetti L, Zsengeller Z, Szabo C, Brownlee M. 2003 Inhibition of GAPDH activity by poly(ADP-ribose) polymerase activates three major pathways of hyperglycemic damage in endothelial cells. J Clin Invest 112: 1049–1057. 14523042 10.1172/JCI18127 PMC198524

[GAD334284SZAC59] El-Hamoly T, Hegedus C, Lakatos P, Kovacs K, Bai P, El-Ghazaly MA, El-Denshary ES, Szabo E, Virag L. 2014 Activation of poly(ADP-ribose) polymerase-1 delays wound healing by regulating keratinocyte migration and production of inflammatory mediators. Mol Med 8: 00130. 10.2119/molmed.2014.00130 PMC4153841 25014793

[GAD334284SZAC60] Erener S, Hesse M, Kostadinova R, Hottiger MO. 2012a Poly(ADP-Ribose)Polymerase-1 (PARP1) controls adipogenic gene expression and adipocyte function. Mol Endocrinol 26: 79–86. 10.1210/me.2011-1163 22053002 PMC5417161

[GAD334284SZAC61] Erener S, Mirsaidi A, Hesse M, Tiaden AN, Ellingsgaard H, Kostadinova R, Donath MY, Richards PJ, Hottiger MO. 2012b *ARTD1* deletion causes increased hepatic lipid accumulation in mice fed a high-fat diet and impairs adipocyte function and differentiation. FASEB J 26: 2631–2638. 10.1096/fj.11-200212 22426118

[GAD334284SZAC62] Fajas L, Auboeuf D, Raspe E, Schoonjans K, Lefebvre AM, Saladin R, Najib J, Laville M, Fruchart JC, Deeb S, 1997 The organization, promoter analysis, and expression of the human PPARγ gene. J Biol Chem 272: 18779–18789. 9228052 10.1074/jbc.272.30.18779

[GAD334284SZAC63] Fajas L, Fruchart JC, Auwerx J. 1998 Transcriptional control of adipogenesis. Curr Opin Cell Biol 10: 165–173. 9561840 10.1016/s0955-0674(98)80138-5

[GAD334284SZAC64] Faraone-Mennella MR, Masi A, Ferreri C. 2019 Regulatory roles of PARP-1 and lipids in epigenetic mechanisms. In Handbook of Nutrition, Diet, and Epigenetics (ed. Patel VB, Preedy VR), pp. 369–386. Springer International Publishing, Cham, Switzerland.

[GAD334284SZAC65] Farrés J, Martín-Caballero J, Martínez C, Lozano JJ, Llacuna L, Ampurdanés C, Ruiz-Herguido C, Dantzer F, Schreiber V, Villunger A, 2013 PARP-2 is required to maintain hematopoiesis following sublethal γ-irradiation in mice. Blood 122: 44–54. 10.1182/blood-2012-12-472845 23678004 PMC4918799

[GAD334284SZAC66] Farrés J, Llacuna L, Martín-Caballero J, Martinez C, Lozano JJ, Ampurdanés C, López-Contreras AJ, Florensa L, Navarro J, Ottina E, 2015 PARP-2 sustains erythropoiesis in mice by limiting replicative stress in erythroid progenitors. Cell Death Differ 22: 1144–1157. 10.1038/cdd.2014.202 25501596 PMC4568570

[GAD334284SZAC67] Fatokun AA, Dawson VL, Dawson TM. 2014 Parthanatos: mitochondrial-linked mechanisms and therapeutic opportunities. Br J Pharmacol 171: 2000–2016. 10.1111/bph.12416 24684389 PMC3976618

[GAD334284SZAC68] Fehr A, Singh SA, Kerr CM, Mukai S, Higashi H, Aikawa M. 2020 The impact of PARPs and ADP-ribosylation on inflammation and host–pathogen interactions. Genes Dev (this issue) 10.1101/gad.334425.119 PMC7050484 32029454

[GAD334284SZAC69] Feijs KL, Forst AH, Verheugd P, Lüscher B. 2013 Macrodomain-containing proteins: regulating new intracellular functions of mono(ADP-ribosyl)ation. Nat Rev Mol Cell Biol 14: 443–451. 10.1038/nrm3601 23736681 PMC7097401

[GAD334284SZAC70] Fouquerel E, Goellner EM, Yu Z, Gagne JP, Barbi de Moura M, Feinstein T, Wheeler D, Redpath P, Li J, Romero G, 2014 ARTD1/PARP1 negatively regulates glycolysis by inhibiting hexokinase 1 independent of NAD depletion. Cell Rep 10: 00712–00718. 10.1016/j.celrep.2014.08.036 PMC4177344 25220464

[GAD334284SZAC71] Gagné JP, Moreel X, Gagné P, Labelle Y, Droit A, Chevalier-Paré M, Bourassa S, McDonald D, Hendzel MJ, Prigent C, 2009 Proteomic investigation of phosphorylation sites in poly(ADP-ribose) polymerase-1 and poly(ADP-ribose) glycohydrolase. J Proteome Res 8: 1014–1029. 10.1021/pr800810n 19105632

[GAD334284SZAC72] Garaulet M, Hernandez-Morante JJ, Lujan J, Tebar FJ, Zamora S. 2006 Relationship between fat cell size and number and fatty acid composition in adipose tissue from different fat depots in overweight/obese humans. Int J Obes (Lond) 30: 899–905. 10.1038/sj.ijo.0803219 16446749

[GAD334284SZAC73] Gariani K, Ryu D, Menzies KJ, Yi HS, Stein S, Zhang H, Perino A, Lemos V, Katsyuba E, Jha P, 2017 Inhibiting poly ADP-ribosylation increases fatty acid oxidation and protects against fatty liver disease. J Hepatol 66: 132–141. 10.1016/j.jhep.2016.08.024 27663419

[GAD334284SZAC74] Gelman L, Zhou G, Fajas L, Raspé E, Fruchart JC, Auwerx J. 1999 p300 interacts with the N- and C-terminal part of PPARγ2 in a ligand-independent and -dependent manner, respectively. J Biol Chem 274: 7681–7688. 10.1074/jbc.274.12.7681 10075656

[GAD334284SZAC75] Ghabreau L, Roux JP, Frappart PO, Mathevet P, Patricot LM, Mokni M, Korbi S, Wang ZQ, Tong WM, Frappart L. 2004 Poly(ADP-ribose) polymerase-1, a novel partner of progesterone receptors in endometrial cancer and its precursors. Int J Cancer 109: 317–321. 10.1002/ijc.11731 14961567

[GAD334284SZAC76] Gibson BA, Kraus WL. 2012 New insights into the molecular and cellular functions of poly(ADP-ribose) and PARPs. Nat Rev Mol Cell Biol 13: 411–424. 10.1038/nrm3376 22713970

[GAD334284SZAC77] Gibson BA, Zhang Y, Jiang H, Hussey KM, Shrimp JH, Lin H, Schwede F, Yu Y, Kraus WL. 2016 Chemical genetic discovery of PARP targets reveals a role for PARP-1 in transcription elongation. Science 353: 45–50. 10.1126/science.aaf7865 27256882 PMC5540732

[GAD334284SZAC78] Gillum MP, Kotas ME, Erion DM, Kursawe R, Chatterjee P, Nead KT, Muise ES, Hsiao JJ, Frederick DW, Yonemitsu S, 2011 SirT1 regulates adipose tissue inflammation. Diabetes 60: 3235–3245. 10.2337/db11-0616 22110092 PMC3219953

[GAD334284SZAC79] Gradwohl G, Menissier de Murcia JM, Molinete M, Simonin F, Koken M, Hoeijmakers JH, de Murcia G. 1990 The second zinc-finger domain of poly(ADP-ribose) polymerase determines specificity for single-stranded breaks in DNA. Proc Natl Acad Sci 87: 2990–2994. 10.1073/pnas.87.8.2990 2109322 PMC53819

[GAD334284SZAC80] Guerriero G, Brundo MV, Labar S, Bianchi AR, Trocchia S, Rabbito D, Palumbo G, Abdel-Gawad FK, De Maio A. 2018 Frog (Pelophylax bergeri, Gunther 1986) endocrine disruption assessment: characterization and role of skin poly(ADP-ribose) polymerases. Environ Sci Pollut Res Int 25: 18303–18313. 10.1007/s11356-017-0395-2 29081042

[GAD334284SZAC81] Gui B, Gui F, Takai T, Feng C, Bai X, Fazli L, Dong X, Liu S, Zhang X, Zhang W, 2019 Selective targeting of PARP-2 inhibits androgen receptor signaling and prostate cancer growth through disruption of FOXA1 function. Proc Natl Acad Sci 116: 14573–14582. 10.1073/pnas.1908547116 31266892 PMC6642419

[GAD334284SZAC82] Hans CP, Zerfaoui M, Naura AS, Catling A, Boulares AH. 2008 Differential effects of PARP inhibition on vascular cell survival and ACAT-1 expression favouring atherosclerotic plaque stability. Cardiovasc Res 78: 429–439. 10.1093/cvr/cvn018 18245064

[GAD334284SZAC83] Hans CP, Feng Y, Naura AS, Zerfaoui M, Rezk BM, Xia H, Kaye AD, Matrougui K, Lazartigues E, Boulares AH. 2009a Protective effects of PARP-1 knockout on dyslipidemia-induced autonomic and vascular dysfunction in ApoE mice: effects on eNOS and oxidative stress. PLoS One 4: e7430 10.1371/journal.pone.0007430 19823587 PMC2757717

[GAD334284SZAC84] Hans CP, Zerfaoui M, Naura AS, Troxclair D, Strong JP, Matrougui K, Boulares AH. 2009b Thieno[2,3-*c*]isoquinolin-5-one, a potent poly(ADP-ribose) polymerase inhibitor, promotes atherosclerotic plaque regression in high-fat diet-fed apolipoprotein E-deficient mice: effects on inflammatory markers and lipid content. J Pharmacol Exp Ther 329: 150–158. 10.1124/jpet.108.145938 19124646 PMC2670599

[GAD334284SZAC85] Hatori M, Vollmers C, Zarrinpar A, DiTacchio L, Bushong EA, Gill S, Leblanc M, Chaix A, Joens M, Fitzpatrick JA, 2012 Time-restricted feeding without reducing caloric intake prevents metabolic diseases in mice fed a high-fat diet. Cell Metab 15: 848–860. 10.1016/j.cmet.2012.04.019 22608008 PMC3491655

[GAD334284SZAC86] Holechek J, Lease R, Thorsell AG, Karlberg T, McCadden C, Grant R, Keen A, Callahan E, Schüler H, Ferraris D. 2018 Design, synthesis and evaluation of potent and selective inhibitors of mono-(ADP-ribosyl)transferases PARP10 and PARP14. Bioorg Med Chem Lett 28: 2050–2054. 10.1016/j.bmcl.2018.04.056 29748053

[GAD334284SZAC87] Hopp AK, Grüter P, Hottiger MO. 2019 Regulation of glucose metabolism by NAD^+^ and ADP-ribosylation. Cells 8: 890 10.3390/cells8080890 PMC6721828 31412683

[GAD334284SZAC88] Horvath EM, Benkő R, Gerő D, Kiss L, Szabó C. 2008 Treatment with insulin inhibits poly(ADP-ribose)polymerase activation in a rat model of endotoxemia. Life Sci 82: 205–209. 10.1016/j.lfs.2007.11.001 18078960 PMC2713048

[GAD334284SZAC89] Hottiger MO. 2015 Nuclear ADP-ribosylation and its role in chromatin plasticity, cell differentiation, and epigenetics. Annu Rev Biochem 84: 227–263. 10.1146/annurev-biochem-060614-034506 25747399

[GAD334284SZAC90] Hottiger MO, Hassa PO, Lüscher B, Schüler H, Koch-Nolte F. 2010 Toward a unified nomenclature for mammalian ADP-ribosyltransferases. Trends Biochem Sci 35: 208–219. 10.1016/j.tibs.2009.12.003 20106667

[GAD334284SZAC91] Houtkooper RH, Cantó C, Wanders RJ, Auwerx J. 2010 The secret life of NAD^+^: an old metabolite controlling new metabolic signaling pathways. Endocr Rev 31: 194–223. 10.1210/er.2009-0026 20007326 PMC2852209

[GAD334284SZAC92] Hu B, Wu Z, Hergert P, Henke CA, Bitterman PB, Phan SH. 2013 Regulation of myofibroblast differentiation by poly(ADP-ribose) polymerase 1. Am J Pathol 182: 71–83. 10.1016/j.ajpath.2012.09.004 23260200 PMC3532707

[GAD334284SZAC93] Huang D, Yang C, Wang Y, Liao Y, Huang K. 2009 PARP-1 suppresses adiponectin expression through poly(ADP-ribosyl)ation of PPARγ in cardiac fibroblasts. Cardiovasc Res 81: 98–107. 10.1093/cvr/cvn264 18815186

[GAD334284SZAC94] Huang K, Du M, Tan X, Yang L, Li X, Jiang Y, Wang C, Zhang F, Zhu F, Cheng M, 2017 PARP1-mediated PPARα poly(ADP-ribosyl)ation suppresses fatty acid oxidation in non-alcoholic fatty liver disease. J Hepatol 66: 962–977. 10.1016/j.jhep.2016.11.020 27979751 PMC9289820

[GAD334284SZAC95] Huang S, Zhang B, Chen Y, Liu H, Liu Y, Li X, Bao Z, Song Z, Wang Z. 2018 Poly(ADP-ribose) polymerase inhibitor PJ34 attenuated hepatic triglyceride accumulation in alcoholic fatty liver disease in mice. J Pharmacol Exp Ther 364: 452–461. 10.1124/jpet.117.243105 29317476

[GAD334284SZAC96] Huang W, Su L, Zhang X, Xu X, Li R. 2019 Endocrinological characterization of pancreatic ducts in HFD and HGD fed mice. J Cell Biochem 120: 16153–16159. 10.1002/jcb.28896 31081956

[GAD334284SZAC97] Hutin D, Tamblyn L, Gomez A, Grimaldi G, Soedling H, Cho T, Ahmed S, Lucas C, Kanduri C, Grant DM, 2018 Hepatocyte-specific deletion of TIPARP, a negative regulator of the aryl hydrocarbon receptor, is sufficient to increase sensitivity to dioxin-induced wasting syndrome. Toxicol Sci 165: 347–360. 10.1093/toxsci/kfy136 29873790 PMC6154274

[GAD334284SZAC98] Idogawa M, Yamada T, Honda K, Sato S, Imai K, Hirohashi S. 2005 Poly(ADP-ribose) polymerase-1 is a component of the oncogenic T-cell factor-4/β-catenin complex. Gastroenterology 128: 1919–1936. 10.1053/j.gastro.2005.03.007 15940627

[GAD334284SZAC99] Javle M, Curtin NJ. 2011 The role of PARP in DNA repair and its therapeutic exploitation. Br J Cancer 105: 1114–1122. 10.1038/bjc.2011.382 21989215 PMC3208503

[GAD334284SZAC100] JAX. 2019 B6(Cg)-Parp1tm1c(EUCOMM)Hmgu/WlkrJ mice. The Jackson Laboratory, https://www.jax.org/strain/032650.

[GAD334284SZAC101] Jog NR, Caricchio R. 2013 Differential regulation of cell death programs in males and females by Poly (ADP-Ribose) Polymerase-1 and 17β estradiol. Cell Death Dis 4: e758 10.1038/cddis.2013.251 23928697 PMC3763428

[GAD334284SZAC102] Joshi A, Mahfooz S, Maurya VK, Kumar V, Basanna CS, Kaur G, Hanif K, Jha RK. 2014 PARP1 during embryo implantation and its upregulation by oestradiol in mice. Reproduction 147: 765–780. 10.1530/REP-13-0588 24516177

[GAD334284SZAC103] Ju BG, Lunyak VV, Perissi V, Garcia-Bassets I, Rose DW, Glass CK, Rosenfeld MG. 2006 A topoisomerase IIβ-mediated dsDNA break required for regulated transcription. Science 312: 1798–1802. 10.1126/science.1127196 16794079

[GAD334284SZAC104] Jukarainen S, Heinonen S, Rämö JT, Rinnankoski-Tuikka R, Rappou E, Tummers M, Muniandy M, Hakkarainen A, Lundbom J, Lundbom N, 2016 Obesity is associated with low NAD^+^/SIRT pathway expression in adipose tissue of BMI-discordant monozygotic twins. J Clin Endocrinol Metab 101: 275–283. 10.1210/jc.2015-3095 26574954

[GAD334284SZAC105] Kajimura S. 2015 Promoting brown and beige adipocyte biogenesis through the PRDM16 pathway. Int J Obes Suppl 5: S11–S14. 10.1038/ijosup.2015.4 27152168 PMC4850573

[GAD334284SZAC106] Karlberg T, Langelier MF, Pascal JM, Schüler H. 2013 Structural biology of the writers, readers, and erasers in mono- and poly(ADP-ribose) mediated signaling. Mol Aspects Med 34: 1088–1108. 10.1016/j.mam.2013.02.002 23458732 PMC3726583

[GAD334284SZAC107] Kawaichi M, Oka J, Zhang J, Ueda K, Hayaishi O. 1983 Properties of poly(ADP-ribose) synthetase and ADP-ribosyl histone splitting enzyme. Princess Takamatsu Symp 13: 121–128. 6317633

[GAD334284SZAC108] Kazak L, Chouchani ET, Lu GZ, Jedrychowski MP, Bare CJ, Mina AI, Kumari M, Zhang S, Vuckovic I, Laznik-Bogoslavski D, 2017 Genetic depletion of adipocyte creatine metabolism inhibits diet-induced thermogenesis and drives obesity. Cell Metab 26: 660–671.e3. 10.1016/j.cmet.2017.08.009 28844881 PMC5629120

[GAD334284SZAC109] Kettner NM, Mayo SA, Hua J, Lee C, Moore DD, Fu L. 2015 Circadian dysfunction induces leptin resistance in mice. Cell Metab 22: 448–459. 10.1016/j.cmet.2015.06.005 26166747 PMC4558341

[GAD334284SZAC110] Khanh VC, Zulkifli AF, Tokunaga C, Yamashita T, Hiramatsu Y, Ohneda O. 2018 Aging impairs beige adipocyte differentiation of mesenchymal stem cells via the reduced expression of Sirtuin 1. Biochem Biophys Res Commun 500: 682–690. 10.1016/j.bbrc.2018.04.136 29678576

[GAD334284SZAC111] Kim KH, Lee MS. 2014 Autophagy as a crosstalk mediator of metabolic organs in regulation of energy metabolism. Rev Endocr Metab Disord 15: 11–20. 10.1007/s11154-013-9272-6 24085381

[GAD334284SZAC112] Kiss L, Chen M, Gero D, Modis K, Lacza Z, Szabo C. 2006 Effects of 7-ketocholesterol on the activity of endothelial poly(ADP-ribose) polymerase and on endothelium-dependent relaxant function. Int J Mol Med 18: 1113–1117. 17089016

[GAD334284SZAC113] Kiss B, Szántó M, Szklenár M, Brunyánszki A, Marosvölgyi T, Sárosi E, Remenyik E, Gergely P, Virág L, Decsi T, 2015 Poly(ADP) ribose polymerase-1 ablation alters eicosanoid and docosanoid signaling and metabolism in a murine model of contact hypersensitivity. Mol Med Rep 11: 2861–2867. 10.3892/mmr.2014.3044 25482287

[GAD334284SZAC114] Kiss B, Szántó M, Hegedűs C, Antal D, Szödényi A, Márton J, Méhes G, Virág L, Szegedi A, Bai P. 2019 Poly(ADP-ribose) polymerase-1 depletion enhances the severity of inflammation in an imiquimod-induced model of psoriasis. Exp Dermatol 10.1111/exd.14061. 31755591

[GAD334284SZAC115] Kleine H, Herrmann A, Lamark T, Forst AH, Verheugd P, Lüscher-Firzlaff J, Lippok B, Feijs KL, Herzog N, Kremmer E, 2012 Dynamic subcellular localization of the mono-ADP-ribosyltransferase ARTD10 and interaction with the ubiquitin receptor p62. Cell Commun Signal 10: 28 10.1186/1478-811X-10-28 22992334 PMC3508985

[GAD334284SZAC116] Koc A, Aydin Sayitoglu M, Karakurt F, Batar B, Niyazoglu M, Celik O, Onaran I, Tasan E, Sultuybek GK. 2014 Association of three SNPs in the PARP-1 gene with Hashimoto's thyroiditis. Hum Genome Var 1: 14016 10.1038/hgv.2014.16 27081507 PMC4785522

[GAD334284SZAC117] Komjati K, Mabley JG, Virag L, Southan GJ, Salzman AL, Szabo C. 2004 Poly(ADP-ribose) polymerase inhibition protect neurons and the white matter and regulates the translocation of apoptosis-inducing factor in stroke. Int J Mol Med 13: 373–382. 14767566

[GAD334284SZAC118] Kraus WL, Hottiger MO. 2013 PARP-1 and gene regulation: progress and puzzles. Mol Aspects Med 34: 1109–1123. 10.1016/j.mam.2013.01.005 23357755

[GAD334284SZAC119] Krishnakumar R, Gamble MJ, Frizzell KM, Berrocal JG, Kininis M, Kraus WL. 2008 Reciprocal binding of PARP-1 and histone H1 at promoters specifies transcriptional outcomes. Science 319: 819–821. 10.1126/science.1149250 18258916

[GAD334284SZAC120] Kristóf E, Doan-Xuan QM, Sárvari AK, Klusóczki A, Fischer-Posovszky P, Wabitsch M, Bacso Z, Bai P, Balajthy Z, Fésus L. 2016 Clozapine modifies the differentiation program of human adipocytes inducing browning. Transl Psychiatry 6: e963 10.1038/tp.2016.230 27898069 PMC5290354

[GAD334284SZAC121] Kutuzov MM, Khodyreva SN, Amé JC, Ilina ES, Sukhanova MV, Schreiber V, Lavrik OI. 2013 Interaction of PARP-2 with DNA structures mimicking DNA repair intermediates and consequences on activity of base excision repair proteins. Biochimie 95: 1208–1215. 10.1016/j.biochi.2013.01.007 23357680

[GAD334284SZAC122] Kutuzov MM, Khodyreva SN, Ilina ES, Sukhanova MV, Amé JC, Lavrik OI. 2015 Interaction of PARP-2 with AP site containing DNA. Biochimie 112: 10–19. 10.1016/j.biochi.2015.02.010 25724268

[GAD334284SZAC123] Larmonier CB, Shehab KW, Laubitz D, Jamwal DR, Ghishan FK, Kiela PR. 2016 Transcriptional reprogramming and resistance to colonic mucosal injury in poly(ADP-ribose) polymerase 1 (PARP1)-deficient mice. J Biol Chem 291: 8918–8930. 10.1074/jbc.M116.714386 26912654 PMC4861461

[GAD334284SZAC124] Léger K, Bär D, Savić N, Santoro R, Hottiger MO. 2014 ARTD2 activity is stimulated by RNA. Nucleic Acids Res 42: 5072–5082. 10.1093/nar/gku131 24510188 PMC4005644

[GAD334284SZAC125] Lehmann M, Pirinen E, Mirsaidi A, Kunze FA, Richards PJ, Auwerx J, Hottiger MO. 2015 ARTD1-induced poly-ADP-ribose formation enhances PPARγ ligand binding and co-factor exchange. Nucleic Acids Res 43: 129–142. 10.1093/nar/gku1260 25452336 PMC4288160

[GAD334284SZAC126] Leslie Pedrioli DM, Leutert M, Bilan V, Nowak K, Gunasekera K, Ferrari E, Imhof R, Malmstrom L, Hottiger MO. 2018 Comprehensive ADP-ribosylome analysis identifies tyrosine as an ADP-ribose acceptor site. EMBO Rep 19: e45310 10.15252/embr.201745310 29954836 PMC6073207

[GAD334284SZAC127] Li B, Li L, Li M, Lam SM, Wang G, Wu Y, Zhang H, Niu C, Zhang X, Liu X, 2019 Microbiota depletion impairs thermogenesis of brown adipose tissue and browning of white adipose tissue. Cell Rep 26: 2720–2737.e5. 10.1016/j.celrep.2019.02.015 30840893

[GAD334284SZAC128] Liu FQ, Zhang XL, Gong L, Wang XP, Wang J, Hou XG, Sun Y, Qin WD, Wei SJ, Zhang Y, 2011 Glucagon-like peptide 1 protects microvascular endothelial cells by inactivating the PARP-1/iNOS/NO pathway. Mol Cell Endocrinol 339: 25–33. 10.1016/j.mce.2011.03.007 21458523

[GAD334284SZAC129] Lönn P, van der Heide LP, Dahl M, Hellman U, Heldin CH, Moustakas A. 2010 PARP-1 attenuates Smad-mediated transcription. Mol Cell 40: 521–532. 10.1016/j.molcel.2010.10.029 21095583

[GAD334284SZAC130] Luche E, Sengenès C, Arnaud E, Laharrague P, Casteilla L, Cousin B. 2015 Differential hematopoietic activity in white adipose tissue depending on its localization. J Cell Physiol 230: 3076–3083. 10.1002/jcp.25045 25989607

[GAD334284SZAC131] Luo X, Ryu KW, Kim DS, Nandu T, Medina CJ, Gupte R, Gibson BA, Soccio RE, Yu Y, Gupta RK, 2017 PARP-1 controls the adipogenic transcriptional program by PARylating C/EBPβ and modulating its transcriptional activity. Mol Cell 65: 260–271. 10.1016/j.molcel.2016.11.015 28107648 PMC5258183

[GAD334284SZAC132] Mabley JG, Horváth EM, Murthy KG, Zsengellér Z, Vaslin A, Benkő R, Kollai M, Szabó C. 2005 Gender differences in the endotoxin-induced inflammatory and vascular responses: potential role of poly(ADP-ribose) polymerase activation. J Pharmacol Exp Ther 315: 812–820. 10.1124/jpet.105.090480 16079296

[GAD334284SZAC133] Mangerich A, Herbach N, Hanf B, Fischbach A, Popp O, Moreno-Villanueva M, Bruns OT, Bürkle A. 2010 Inflammatory and age-related pathologies in mice with ectopic expression of human PARP-1. Mech Ageing Dev 131: 389–404. 10.1016/j.mad.2010.05.005 20561897

[GAD334284SZAC134] Manunza A, Casellas J, Quintanilla R, González-Prendes R, Pena RN, Tibau J, Mercade A, Castelló A, Aznárez N, Hernández-Sánchez J, 2014 A genome-wide association analysis for porcine serum lipid traits reveals the existence of age-specific genetic determinants. BMC Genomics 15: 758 10.1186/1471-2164-15-758 25189197 PMC4164741

[GAD334284SZAC135] Mariotti L, Pollock K, Guettler S. 2017 Regulation of Wnt/β-catenin signalling by tankyrase-dependent poly(ADP-ribosyl)ation and scaffolding. Br J Pharmacol 174: 4611–4636. 10.1111/bph.14038 28910490 PMC5727255

[GAD334284SZAC136] Marques M, Jangal M, Wang LC, Kazanets A, da Silva SD, Zhao T, Lovato A, Yu H, Jie S, Del Rincon S, 2019 Oncogenic activity of poly (ADP-ribose) glycohydrolase. Oncogene 38: 2177–2191. 10.1038/s41388-018-0568-6 30459355 PMC6484711

[GAD334284SZAC137] Martinet W, Knaapen MW, De Meyer GR, Herman AG, Kockx MM. 2002 Elevated levels of oxidative DNA damage and DNA repair enzymes in human atherosclerotic plaques. Circulation 106: 927–932. 10.1161/01.CIR.0000026393.47805.21 12186795

[GAD334284SZAC138] Márton J, Fodor T, Nagy L, Vida A, Kis G, Brunyánszki A, Antal M, Lüscher B, Bai P. 2018a PARP10 (ARTD10) modulates mitochondrial function. PLoS One 13: e0187789 10.1371/journal.pone.0187789 29293500 PMC5749700

[GAD334284SZAC139] Marton J, Peter M, Balogh G, Bodi B, Vida A, Szanto M, Bojcsuk D, Janko L, Bhattoa HP, Gombos I, 2018b Poly(ADP-ribose) polymerase-2 is a lipid-modulated modulator of muscular lipid homeostasis. Biochim Biophys Acta 2: 30187–30182. 10.1016/j.bbalip.2018.07.013 30077797

[GAD334284SZAC140] Masszi G, Horvath EM, Tarszabo R, Benko R, Novak A, Buday A, Tokes AM, Nadasy GL, Hamar P, Benyó Z, 2013 Reduced estradiol-induced vasodilation and poly-(ADP-ribose) polymerase (PARP) activity in the aortas of rats with experimental polycystic ovary syndrome (PCOS). PLoS One 8: e55589 10.1371/journal.pone.0055589 23555555 PMC3608629

[GAD334284SZAC141] Menissier-de Murcia J, Molinete M, Gradwohl G, Simonin F, de Murcia G. 1989 Zinc-binding domain of poly(ADP-ribose)polymerase participates in the recognition of single strand breaks on DNA. J Mol Biol 210: 229–233. 2511329 10.1016/0022-2836(89)90302-1

[GAD334284SZAC142] Miyamoto T, Kakizawa T, Hashizume K. 1999 Inhibition of nuclear receptor signalling by poly(ADP-ribose) polymerase. Mol Cell Biol 19: 2644–2649. 10082530 10.1128/mcb.19.4.2644 PMC84057

[GAD334284SZAC143] Mocchegiani E, Muzzioli M, Giacconi R, Cipriano C, Gasparini N, Franceschi C, Gaetti R, Cavalieri E, Suzuki H. 2003 Metallothioneins/PARP-1/IL-6 interplay on natural killer cell activity in elderly: parallelism with nonagenarians and old infected humans. Effect of zinc supply. Mech Ageing Dev 124: 459–468. 10.1016/S0047-6374(03)00023-X 12714254

[GAD334284SZAC144] Mocchegiani E, Giacconi R, Cipriano C, Gasparini N, Bernardini G, Malavolta M, Menegazzi M, Cavalieri E, Muzzioli M, Ciampa AR, 2004 The variations during the circadian cycle of liver CD1d-unrestricted NK1.1^+^ TCRγ/δ^+^ cells lead to successful ageing. Role of metallothionein/IL-6/gp130/PARP-1 interplay in very old mice. Exp Gerontol 39: 775–788. 10.1016/j.exger.2004.01.014 15130672

[GAD334284SZAC145] Módis K, Gerő D, Erdélyi K, Szoleczky P, Dewitt D, Szabo C. 2012 Cellular bioenergetics is regulated by PARP1 under resting conditions and during oxidative stress. Biochem Pharmacol 83: 633–643. 10.1016/j.bcp.2011.12.014 22198485 PMC3272837

[GAD334284SZAC146] Mohamed JS, Hajira A, Pardo PS, Boriek AM. 2014 MicroRNA-149 inhibits PARP-2 and promotes mitochondrial biogenesis via SIRT-1/PGC-1α network in skeletal muscle. Diabetes 63: 1546–1559. 10.2337/db13-1364 24757201

[GAD334284SZAC147] Morrow DA, Brickman CM, Murphy SA, Baran K, Krakover R, Dauerman H, Kumar S, Slomowitz N, Grip L, McCabe CH, 2009 A randomized, placebo-controlled trial to evaluate the tolerability, safety, pharmacokinetics, and pharmacodynamics of a potent inhibitor of poly(ADP-ribose) polymerase (INO-1001) in patients with ST-elevation myocardial infarction undergoing primary percutaneous coronary intervention: results of the TIMI 37 trial. J Thromb Thrombolysis 27: 359–364. 10.1007/s11239-008-0230-1 18535785

[GAD334284SZAC148] Mota de Sa P, Richard AJ, Hang H, Stephens JM. 2017 Transcriptional regulation of adipogenesis. Compr Physiol 7: 635–674. 28333384 10.1002/cphy.c160022

[GAD334284SZAC149] Muiras ML, Müller M, Schächter F, Bürkle A. 1998 Increased poly(ADP-ribose) polymerase activity in lymphoblastoid cell lines from centenarians. J Mol Med (Berl) 76: 346–354. 10.1007/s001090050226 9587069

[GAD334284SZAC150] Mukhopadhyay P, Horváth B, Rajesh M, Varga ZV, Gariani K, Ryu D, Cao Z, Holovac E, Park O, Zhou Z, 2017 PARP inhibition protects against alcoholic and non-alcoholic steatohepatitis. J Hepatol 66: 589–600. 10.1016/j.jhep.2016.10.023 27984176

[GAD334284SZAC151] Muñoz-Gámez JA, Rodríguez-Vargas JM, Quiles-Perez R, Aguilar-Quesada R, Martin-Oliva D, de Murcia G, de Murcia Murcia J, Almendros A, Ruiz de Almodóvar M, Oliver FJ. 2009 PARP-1 is involved in autophagy induced by DNA damage. Autophagy 5: 61–74. 10.4161/auto.5.1.7272 19001878

[GAD334284SZAC152] Muthumani K, Choo AY, Zong WX, Madesh M, Hwang DS, Premkumar A, Thieu KP, Emmanuel J, Kumar S, Thompson CB, 2006 The HIV-1 Vpr and glucocorticoid receptor complex is a gain-of-function interaction that prevents the nuclear localization of PARP-1. Nat Cell Biol 8: 170–179. 10.1038/ncb1352 16429131 PMC3142937

[GAD334284SZAC153] Nagy L, Tontonoz P, Alvarez JG, Chen H, Evans RM. 1998 Oxidized LDL regulates macrophage gene expression through ligand activation of PPARγ. Cell 93: 229–240. 10.1016/S0092-8674(00)81574-3 9568715

[GAD334284SZAC154] Nagy L, Rauch B, Balla N, Ujlaki G, Kis G, Abdul-Rahman O, Kristof E, Sipos A, Antal M, Toth A, 2019 Olaparib induces browning of in vitro cultures of human primary white adipocytes. Biochem Pharmacol 25: 30250–30253. 10.1016/j.bcp.2019.06.022 31251940

[GAD334284SZAC155] Nomura F, Yaguchi M, Itoga And S, Noda M. 2001 Effects of chronic alcohol consumption on hepatic poly-ADP-ribosylation in the rat. Alcohol Clin Exp Res 25: 35S–38S. 10.1111/j.1530-0277.2001.tb02415.x 11410739

[GAD334284SZAC156] Nozaki T, Fujihara H, Watanabe M, Tsutsumi M, Nakamoto K, Kusuoka O, Kamada N, Suzuki H, Nakagama H, Sugimura T, 2003 Parp-1 deficiency implicated in colon and liver tumorigenesis induced by azoxymethane. Cancer Sci 94: 497–500. 10.1111/j.1349-7006.2003.tb01472.x 12824873 PMC11160212

[GAD334284SZAC157] Nozaki T, Fujimori H, Wang J, Suzuki H, Imai H, Watanabe M, Ohura K, Masutani M. 2013 *Parp-1* deficiency in ES cells promotes invasive and metastatic lesions accompanying induction of trophoblast giant cells during tumorigenesis in uterine environment. Pathol Int 63: 408–414. 10.1111/pin.12086 23957916

[GAD334284SZAC158] Ohkura N, Nagamura Y, Tsukada T. 2008 Differential transactivation by orphan nuclear receptor NOR1 and its fusion gene product EWS/NOR1: possible involvement of poly(ADP-ribose) polymerase I, PARP-1. J Cell Biochem 105: 785–800. 10.1002/jcb.21876 18680143

[GAD334284SZAC159] Oka S, Kato J, Moss J. 2006 Identification and characterization of a mammalian 39-kDa poly(ADP-ribose) glycohydrolase. J Biol Chem 281: 705–713. 10.1074/jbc.M510290200 16278211

[GAD334284SZAC160] Oliver FJ, Ménissier-de Murcia J, Nacci C, Decker P, Andriantsitohaina R, Muller S, de La RG, Stoclet JC, de Murcia G. 1999 Resistance to endotoxic shock as a consequence of defective NF-κB activation in poly (ADP-ribose) polymerase-1 deficient mice. EMBO J 18: 4446–4454. 10.1093/emboj/18.16.4446 10449410 PMC1171519

[GAD334284SZAC161] O'Sullivan J, Tedim Ferreira M, Gagné JP, Sharma AK, Hendzel MJ, Masson JY, Poirier GG. 2019 Emerging roles of eraser enzymes in the dynamic control of protein ADP-ribosylation. Nat Commun 10: 1182 10.1038/s41467-019-08859-x 30862789 PMC6414514

[GAD334284SZAC162] Oumouna M, Datta R, Oumouna-Benachour K, Suzuki Y, Hans C, Matthews K, Fallon K, Boulares H. 2006 Poly(ADP-ribose) polymerase-1 inhibition prevents eosinophil recruitment by modulating Th2 cytokines in a murine model of allergic airway inflammation: a potential specific effect on IL-5. J Immunol 177: 6489–6496. 10.4049/jimmunol.177.9.6489 17056581

[GAD334284SZAC163] Oumouna-Benachour K, Hans CP, Suzuki Y, Naura A, Datta R, Belmadani S, Fallon K, Woods C, Boulares AH. 2007 Poly(ADP-ribose) polymerase inhibition reduces atherosclerotic plaque size and promotes factors of plaque stability in apolipoprotein E-deficient mice: effects on macrophage recruitment, nuclear factor-κB nuclear translocation, and foam cell death. Circulation 115: 2442–2450. 10.1161/CIRCULATIONAHA.106.668756 17438151

[GAD334284SZAC164] Pacher P, Szabo C. 2005 Role of poly(ADP-ribose) polymerase-1 activation in the pathogenesis of diabetic complications: endothelial dysfunction, as a common underlying theme. Antioxid Redox Signal 7: 1568–1580. 16356120 10.1089/ars.2005.7.1568 PMC2228261

[GAD334284SZAC165] Pacher P, Szabo C. 2006 Role of peroxynitrite in the pathogenesis of cardiovascular complications of diabetes. Curr Opin Pharmacol 6: 136–141. 16483848 10.1016/j.coph.2006.01.001 PMC2228269

[GAD334284SZAC166] Palazzo L, Leidecker O, Prokhorova E, Dauben H, Matic I, Ahel I. 2018 Serine is the major residue for ADP-ribosylation upon DNA damage. Elife 7 10.7554/eLife.34334 PMC5837557 29480802

[GAD334284SZAC167] Pavri R, Lewis B, Kim TK, Dilworth FJ, Erdjument-Bromage H, Tempst P, de Murcia G, Evans R, Chambon P, Reinberg D. 2005 PARP-1 determines specificity in a retinoid signaling pathway via direct modulation of mediator. Mol Cell 18: 83–96. 15808511 10.1016/j.molcel.2005.02.034

[GAD334284SZAC168] Peng H, Zhu QS, Zhong S, Levy D. 2015 Transcription of the human microsomal epoxide hydrolase gene (EPHX1) Is regulated by PARP-1 and Histone H1.2. Association with sodium-dependent bile acid transport. PLoS One 10: e0125318 10.1371/journal.pone.0125318 25992604 PMC4439041

[GAD334284SZAC169] Pirinen E, Cantó E, Jo SK, Morato L, Zhang H, Menzies KJ, Williams EG, Mouchiroud L, Moullan N, Hagberg C, 2014 Pharmacological inhibition of Poly(ADP-Ribose) polymerases improves fitness and mitochondrial function in skeletal muscle. Cell Metab 19: 1034–1041. 10.1016/j.cmet.2014.04.002 24814482 PMC4047186

[GAD334284SZAC170] Qiang L, Wang L, Kon N, Zhao W, Lee S, Zhang Y, Rosenbaum M, Zhao Y, Gu W, Farmer SR, 2012 Brown remodeling of white adipose tissue by SirT1-dependent deacetylation of Pparγ. Cell 150: 620–632. 10.1016/j.cell.2012.06.027 22863012 PMC3413172

[GAD334284SZAC171] Rack JGM, Palazzo L, Ahel I. 2020 (ADP-ribosyl)hydrolases: structure, function, and biology. Genes Dev (this issue) 10.1101/gad.334631.119 PMC7050489 32029451

[GAD334284SZAC172] Rappou E, Jukarainen S, Rinnankoski-Tuikka R, Kaye S, Heinonen S, Hakkarainen A, Lundbom J, Lundbom N, Saunavaara V, Rissanen A, 2016 Weight loss is associated with increased NAD^+^/SIRT1 expression but reduced PARP activity in white adipose tissue. J Clin Endocrinol Metab 101: 1263–1273. 10.1210/jc.2015-3054 26760174

[GAD334284SZAC173] Regdon Z, Robaszkiewicz A, Kovács K, Rygielska Z, Hegedűs C, Bodoor K, Szabó E, Virág L. 2019 LPS protects macrophages from AIF-independent parthanatos by downregulation of PARP1 expression, induction of SOD2 expression, and a metabolic shift to aerobic glycolysis. Free Radic Biol Med 131: 184–196. 10.1016/j.freeradbiomed.2018.11.034 30502458

[GAD334284SZAC174] Reilly SM, Saltiel AR. 2017 Adapting to obesity with adipose tissue inflammation. Nat Rev Endocrinol 13: 633–643. 10.1038/nrendo.2017.90 28799554

[GAD334284SZAC175] Robaszkiewicz A, Valkó Z, Kovács K, Hegedűs C, Bakondi E, Bai P, Virág L. 2014 The role of p38 signaling and poly(ADP-ribosyl)ation-induced metabolic collapse in the osteogenic differentiation-coupled cell death pathway. Free Radic Biol Med 76C: 69–79. 10.1016/j.freeradbiomed.2014.07.027 25078118

[GAD334284SZAC176] Roca-Rivada A, Alonso J, Al-Massadi O, Castelao C, Peinado JR, Seoane LM, Casanueva FF, Pardo M. 2011 Secretome analysis of rat adipose tissues shows location-specific roles for each depot type. J Proteomics 74: 1068–1079. 10.1016/j.jprot.2011.03.010 21439414

[GAD334284SZAC177] Roper SJ, Chrysanthou S, Senner CE, Sienerth A, Gnan S, Murray A, Masutani M, Latos P, Hemberger M. 2014 ADP-ribosyltransferases Parp1 and Parp7 safeguard pluripotency of ES cells. Nucleic Acids Res 42: 8914–8927. 10.1093/nar/gku591 25034692 PMC4132717

[GAD334284SZAC178] Rosen ED, Spiegelman BM. 2014 What we talk about when we talk about fat. Cell 156: 20–44. 10.1016/j.cell.2013.12.012 24439368 PMC3934003

[GAD334284SZAC179] Ruiz-Ojeda FJ, Rupérez AI, Gomez-Llorente C, Gil A, Aguilera CM. 2016 Cell models and their application for studying adipogenic differentiation in relation to obesity: a review. Int J Mol Sci 17: 1040 10.3390/ijms17071040 PMC4964416 27376273

[GAD334284SZAC180] Ryu KW, Nandu T, Kim J, Challa S, DeBerardinis RJ, Kraus WL. 2018 Metabolic regulation of transcription through compartmentalized NAD^+^ biosynthesis. Science 360: eaan5780 10.1126/science.aan5780 29748257 PMC6465534

[GAD334284SZAC181] Sacks HS, Fain JN, Bahouth SW, Ojha S, Frontini A, Budge H, Cinti S, Symonds ME. 2013 Adult epicardial fat exhibits beige features. J Clin Endocrinol Metab 98: E1448–E1455. 10.1210/jc.2013-1265 23824424

[GAD334284SZAC182] Salomone F, Barbagallo I, Godos J, Lembo V, Currenti W, Cinà D, Avola R, D'Orazio N, Morisco F, Galvano F, 2017 Silibinin restores NAD^+^ levels and induces the SIRT1/AMPK pathway in non-alcoholic fatty liver. Nutrients 9: 1086 10.3390/nu9101086 PMC5691703 28973994

[GAD334284SZAC183] Sanchez-Gurmaches J, Guertin DA. 2014a Adipocyte lineages: tracing back the origins of fat. Biochim Biophys Acta 1842: 340–351. 10.1016/j.bbadis.2013.05.027 23747579 PMC3805734

[GAD334284SZAC184] Sanchez-Gurmaches J, Guertin DA. 2014b Adipocytes arise from multiple lineages that are heterogeneously and dynamically distributed. Nat Commun 5: 4099 10.1038/ncomms5099 24942009 PMC4066194

[GAD334284SZAC185] Scheja L, Heeren J. 2019 The endocrine function of adipose tissues in health and cardiometabolic disease. Nat Rev Endocrinol 15: 507–524. 10.1038/s41574-019-0230-6 31296970

[GAD334284SZAC186] Schiewer MJ, Goodwin JF, Han S, Brenner JC, Augello MA, Dean JL, Liu F, Planck JL, Ravindranathan P, Chinnaiyan AM, 2012 Dual roles of PARP-1 promote cancer growth and progression. Cancer Discov 2012: 19. 10.1158/2159-8290.CD-12-0120 PMC3519969 22993403

[GAD334284SZAC187] Schreiber V, Amé JC, Dollé P, Schultz I, Rinaldi B, Fraulob V, Ménissier-de Murcia J, de Murcia G. 2002 Poly(ADP-ribose) polymerase-2 (PARP-2) is required for efficient base excision DNA repair in association with PARP-1 and XRCC1. J Biol Chem 277: 23028–23036. 10.1074/jbc.M202390200 11948190

[GAD334284SZAC188] Shan T, Liang X, Bi P, Kuang S. 2013 Myostatin knockout drives browning of white adipose tissue through activating the AMPK–PGC1α–Fndc5 pathway in muscle. FASEB J 27: 1981–1989. 10.1096/fj.12-225755 23362117 PMC3633817

[GAD334284SZAC189] Shen X, Wang W, Wang L, Houde C, Wu W, Tudor M, Thompson JR, Sisk CM, Hubbard B, Li J. 2012 Identification of genes affecting apolipoprotein B secretion following siRNA-mediated gene knockdown in primary human hepatocytes. Atherosclerosis 222: 154–157. 10.1016/j.atherosclerosis.2012.02.012 22398276

[GAD334284SZAC190] Shi L, Ko S, Kim S, Echchgadda I, Oh TS, Song CS, Chatterjee B. 2008 Loss of androgen receptor in aging and oxidative stress through Myb protooncoprotein-regulated reciprocal chromatin dynamics of p53 and poly(ADP-ribose) polymerase PARP-1. J Biol Chem 283: 36474–36485. 10.1074/jbc.M805980200 18945670 PMC2606006

[GAD334284SZAC191] Shimizu T, Macey TA, Quillinan N, Klawitter J, Perraud AL, Traystman RJ, Herson PS. 2013 Androgen and PARP-1 regulation of TRPM2 channels after ischemic injury. J Cereb Blood Flow Metab 33: 1549–1555. 10.1038/jcbfm.2013.105 23801245 PMC3790922

[GAD334284SZAC192] Shrestha E, Hussein MA, Savas JN, Ouimet M, Barrett TJ, Leone S, Yates JR III, Moore KJ, Fisher EA, Garabedian MJ. 2016 Poly(ADP-ribose) polymerase 1 represses liver X receptor-mediated ABCA1 expression and cholesterol efflux in macrophages. J Biol Chem 291: 11172–11184. 10.1074/jbc.M116.726729 27026705 PMC4900266

[GAD334284SZAC193] Slade D. 2020 PARP and PARG inhibitors in cancer treatment. Genes Dev (this issue) 10.1101/gad.334516.119 PMC7050487 32029455

[GAD334284SZAC194] Smulson ME, Kang VH, Ntambi JM, Rosenthal DS, Ding R, Simbulan CM. 1995 Requirement for the expression of poly(ADP-ribose) polymerase during the early stages of differentiation of 3T3-L1 preadipocytes, as studied by antisense RNA induction. J Biol Chem 270: 119–127. 7814362 10.1074/jbc.270.1.119

[GAD334284SZAC195] Soriano FG, Pacher P, Mabley J, Liaudet L, Szabo C. 2001 Rapid reversal of the diabetic endothelial dysfunction by pharmacological inhibition of poly(ADP-ribose) polymerase. Circ Res 89: 684–691. 11597991 10.1161/hh2001.097797

[GAD334284SZAC196] Suárez-Zamorano N, Fabbiano S, Chevalier C, Stojanović O, Colin DJ, Stevanović A, Veyrat-Durebex C, Tarallo V, Rigo D, Germain S, 2015 Microbiota depletion promotes browning of white adipose tissue and reduces obesity. Nat Med 21: 1497–1501. 10.1038/nm.3994 26569380 PMC4675088

[GAD334284SZAC197] Suh SW, Aoyama K, Matsumori Y, Liu J, Swanson RA. 2005 Pyruvate administered after severe hypoglycemia reduces neuronal death and cognitive impairment. Diabetes 54: 1452–1458. 10.2337/diabetes.54.5.1452 15855333

[GAD334284SZAC198] Sun K, Gao Z, Kolonin MG. 2018 Transient inflammatory signaling promotes beige adipogenesis. Sci Signal 11: eaat3192 10.1126/scisignal.aat3192 29692362

[GAD334284SZAC199] Sun Q, Gatie M II, Kelly GM. 2019 Serum-dependent and independent regulation of PARP2. Biochem Cell Biol 97: 600–611. 10.1139/bcb-2018-0345 30880404

[GAD334284SZAC200] Sunderland PT, Woon EC, Dhami A, Bergin AB, Mahon MF, Wood PJ, Jones LA, Tully SR, Lloyd MD, Thompson AS, 2011 5-Benzamidoisoquinolin-1-ones and 5-(ω-carboxyalkyl)isoquinolin-1-ones as isoform-selective inhibitors of poly(ADP-ribose) polymerase 2 (PARP-2). J Med Chem 54: 2049–2059. 10.1021/jm1010918 21417348

[GAD334284SZAC201] Szabo C. 2005 Roles of poly(ADP-ribose) polymerase activation in the pathogenesis of diabetes mellitus and its complications. Pharmacol Res 52: 60–71. 15911334 10.1016/j.phrs.2005.02.015

[GAD334284SZAC202] Szabo C, Biser A, Benko R, Bottinger E, Susztak K. 2006 Poly(ADP-ribose) polymerase inhibitors ameliorate nephropathy of type 2 diabetic Leprdb/db mice. Diabetes 55: 3004–3012. 10.2337/db06-0147 17065336

[GAD334284SZAC203] Szanto M, Rutkai I, Hegedűs C, Czikora A, Rózsahegyi M, Kiss B, Virág L, Gergely P, Tóth A, Bai P. 2011 Poly(ADP-ribose) polymerase-2 depletion reduces doxorubicin-induced damage through SIRT1 induction. Cardiovasc Res 92: 430–438. 10.1093/cvr/cvr246 21921080

[GAD334284SZAC204] Szántó M, Brunyánszki A, Kiss B, Nagy L, Gergely P, Virág L, Bai P. 2012 Poly(ADP-ribose) polymerase-2: emerging transcriptional roles of a DNA repair protein. Cell Mol Life Sci 69: 4079–4092. 10.1007/s00018-012-1003-8 22581363 PMC11114944

[GAD334284SZAC205] Szántó M, Brunyánszki A, Márton J, Vámosi G, Nagy L, Fodor T, Kiss B, Virag L, Gergely P, Bai P. 2014 Deletion of PARP-2 induces hepatic cholesterol accumulation and decrease in HDL levels. Biochem Biophys Acta 1842: 594–602. 10.1016/j.bbadis.2013.12.006 24365238

[GAD334284SZAC206] Upton K, Meyers M, Thorsell AG, Karlberg T, Holechek J, Lease R, Schey G, Wolf E, Lucente A, Schüler H, 2017 Design and synthesis of potent inhibitors of the mono(ADP-ribosyl)transferase, PARP14. Bioorg Med Chem Lett 27: 2907–2911. 10.1016/j.bmcl.2017.04.089 28495083

[GAD334284SZAC207] Venkannagari H, Verheugd P, Koivunen J, Haikarainen T, Obaji E, Ashok Y, Narwal M, Pihlajaniemi T, Lüscher B, Lehtiö L. 2016 Small-molecule chemical probe rescues cells from Mono-ADP-ribosyltransferase ARTD10/PARP10-induced apoptosis and sensitizes cancer cells to DNA damage. Cell Chem Biol 23: 1251–1260. 10.1016/j.chembiol.2016.08.012 27667561

[GAD334284SZAC208] Vida A, Abdul-Rahman O, Mikó E, Brunyanszki A, Bai P. 2016 Poly(ADP-Ribose) polymerases in aging—friend or foe? Curr Protein Pept Sci 17: 705–712. 10.2174/1389203717666160419144959 27090903

[GAD334284SZAC209] Vida A, Kardos G, Kovacs T, Bodrogi BL, Bai P. 2018 Deletion of poly(ADPribose) polymerase-1 changes the composition of the microbiome in the gut. Mol Med Rep 18: 4335–4341. 30221733 10.3892/mmr.2018.9474 PMC6172391

[GAD334284SZAC210] Virag L, Salzman AL, Szabo C. 1998a Poly(ADP-ribose) synthetase activation mediates mitochondrial injury during oxidant-induced cell death. J Immunol 161: 3753–3759. 9759901

[GAD334284SZAC211] Virág L, Scott GS, Cuzzocrea S, Marmer D, Salzman AL, Szabó C. 1998b Peroxynitrite-induced thymocyte apoptosis: the role of caspases and poly (ADP-ribose) synthetase (PARS) activation. Immunology 94: 345–355. 10.1046/j.1365-2567.1998.00534.x 9767416 PMC1364252

[GAD334284SZAC212] Vishvanath L, Gupta RK. 2019 Contribution of adipogenesis to healthy adipose tissue expansion in obesity. J Clin Invest 129: 4022–4031. 10.1172/JCI129191 31573549 PMC6763245

[GAD334284SZAC213] Vivelo CA, Wat R, Agrawal C, Tee HY, Leung AKL. 2017 ADPriboDB: the database of ADP-ribosylated proteins. Nucleic Acids Res 45: 6254 10.1093/nar/gkw706 28100694 PMC5449541

[GAD334284SZAC214] von Lukowicz T, Hassa PO, Lohmann C, Borén J, Braunersreuther V, Mach F, Odermatt B, Gersbach M, Camici GG, Stähli BE, 2008 PARP1 is required for adhesion molecule expression in atherogenesis. Cardiovasc Res 78: 158–166. 10.1093/cvr/cvm110 18093987

[GAD334284SZAC215] Vyas DR, McCarthy JJ, Tsika GL, Tsika RW. 2001 Multiprotein complex formation at the β myosin heavy chain distal muscle CAT element correlates with slow muscle expression but not mechanical overload responsiveness. J Biol Chem 276: 1173–1184. 10.1074/jbc.M007750200 11010974

[GAD334284SZAC216] Wacker DA, Ruhl DD, Balagamwala EH, Hope KM, Zhang T, Kraus WL. 2007 The DNA binding and catalytic domains of poly(ADP-ribose) polymerase 1 cooperate in the regulation of chromatin structure and transcription. Mol Cell Biol 27: 7475–7485. 17785446 10.1128/MCB.01314-07 PMC2169059

[GAD334284SZAC217] Wahlberg E, Karlberg T, Kouznetsova E, Markova N, Macchiarulo A, Thorsell AG, Pol E, Frostell A, Ekblad T, Öncu D, 2012 Family-wide chemical profiling and structural analysis of PARP and tankyrase inhibitors. Nat Biotechnol 30: 283–288. 10.1038/nbt.2121 22343925

[GAD334284SZAC218] Wang ZQ, Auer B, Stingl L, Berghammer H, Haidacher D, Schweiger M, Wagner EF. 1995 Mice lacking ADPRT and poly(ADP-ribosyl)ation develop normally but are susceptible to skin disease. Genes Dev 9: 509–520. 10.1101/gad.9.5.509 7698643

[GAD334284SZAC219] Wang S, Liang X, Yang Q, Fu X, Rogers CJ, Zhu M, Rodgers BD, Jiang Q, Dodson MV, Du M. 2015 Resveratrol induces brown-like adipocyte formation in white fat through activation of AMP-activated protein kinase (AMPK) α1. Int J Obes (Lond) 39: 967–976. 10.1038/ijo.2015.23 25761413 PMC4575949

[GAD334284SZAC220] Wang XB, Cui NH, Zhang S, Guo SR, Liu ZJ, Ming L. 2017 PARP-1 Variant Rs1136410 confers protection against coronary artery disease in a Chinese Han population: a two-stage case-control study involving 5643 subjects. Front Physiol 8: 916 10.3389/fphys.2017.00916 29184509 PMC5694467

[GAD334284SZAC221] Wei SJ, Xing JH, Wang BL, Xue L, Wang JL, Li R, Qin WD, Wang J, Wang XP, Zhang MX, 2013 Poly(ADP-ribose) polymerase inhibition prevents reactive oxygen species induced inhibition of aldehyde dehydrogenase2 activity. Biochim Biophys Acta 1833: 479–486. 10.1016/j.bbamcr.2012.11.007 23159776

[GAD334284SZAC222] Wu J, Boström P, Sparks LM, Ye L, Choi JH, Giang AH, Khandekar M, Virtanen KA, Nuutila P, Schaart G, 2012 Beige adipocytes are a distinct type of thermogenic fat cell in mouse and human. Cell 150: 366–376. 10.1016/j.cell.2012.05.016 22796012 PMC3402601

[GAD334284SZAC223] Wu X, Dong Z, Wang CJ, Barlow LJ, Fako V, Serrano MA, Zou Y, Liu JY, Zhang JT. 2016 FASN regulates cellular response to genotoxic treatments by increasing PARP-1 expression and DNA repair activity via NF-κB and SP1. Proc Natl Acad Sci 113: E6965–E6973. 10.1073/pnas.1609934113 27791122 PMC5111708

[GAD334284SZAC224] Xu S, Bai P, Little PJ, Liu P. 2014 Poly(ADP-ribose) polymerase 1 (PARP1) in atherosclerosis: from molecular mechanisms to therapeutic implications. Med Res Rev 34: 644–675. 10.1002/med.21300 24002940

[GAD334284SZAC225] Yamaguchi S, Franczyk MP, Chondronikola M, Qi N, Gunawardana SC, Stromsdorfer KL, Porter LC, Wozniak DF, Sasaki Y, Rensing N, 2019 Adipose tissue NAD^+^ biosynthesis is required for regulating adaptive thermogenesis and whole-body energy homeostasis in mice. Proc Natl Acad Sci 116: 23822–23828. 10.1073/pnas.1909917116 31694884 PMC6876243

[GAD334284SZAC226] Yanagawa T, Funasaka T, Tsutsumi S, Hu H, Watanabe H, Raz A. 2007 Regulation of phosphoglucose isomerase/autocrine motility factor activities by the poly(ADP-ribose) polymerase family-14. Cancer Res 67: 8682–8689. 10.1158/0008-5472.CAN-07-1586 17875708

[GAD334284SZAC227] Ye DZ, Tai MH, Linning KD, Szabo C, Olson LK. 2006 MafA expression and insulin promoter activity are induced by nicotinamide and related compounds in INS-1 pancreatic β-cells. Diabetes 55: 742–750. 10.2337/diabetes.55.03.06.db05-0653 16505238

[GAD334284SZAC228] Yeh TY, Sbodio JI, Tsun ZY, Luo B, Chi NW. 2007 Insulin-stimulated exocytosis of GLUT4 is enhanced by IRAP and its partner tankyrase. Biochem J 402: 279–290. 10.1042/BJ20060793 17059388 PMC1798437

[GAD334284SZAC229] Yeh TY, Beiswenger KK, Li P, Bolin KE, Lee RM, Tsao TS, Murphy AN, Hevener AL, Chi NW. 2009 Hypermetabolism, hyperphagia, and reduced adiposity in tankyrase-deficient mice. Diabetes 11: 2476–2485. 10.2337/db08-1781 PMC2768175 19651815

[GAD334284SZAC230] Yélamos J, Monreal Y, Saenz L, Aguado E, Schreiber V, Mota R, Fuente T, Minguela A, Parrilla P, de Murcia G, 2006 PARP-2 deficiency affects the survival of CD4^+^CD8^+^ double-positive thymocytes. EMBO J 25: 4350–4360. 10.1038/sj.emboj.7601301 16946705 PMC1570435

[GAD334284SZAC231] Ying W, Chen Y, Alano CC, Swanson RA. 2002 Tricarboxylic acid cycle substrates prevent PARP-mediated death of neurons and astrocytes. J Cereb Blood Flow Metab 22: 774–779. 10.1097/00004647-200207000-00002 12142562

[GAD334284SZAC232] Ying W, Garnier P, Swanson RA. 2003 NAD^+^ repletion prevents PARP-1-induced glycolytic blockade and cell death in cultured mouse astrocytes. Biochem Biophys Res Commun 308: 809–813. 10.1016/S0006-291X(03)01483-9 12927790

[GAD334284SZAC233] Zaremba T, Thomas HD, Cole M, Coulthard SA, Plummer ER, Curtin NJ. 2011 Poly(ADP-ribose) polymerase-1 (PARP-1) pharmacogenetics, activity and expression analysis in cancer patients and healthy volunteers. Biochem J 436: 671–679. 10.1042/BJ20101723 21434873

[GAD334284SZAC234] Zarrinpar A, Chaix A, Panda S. 2016 Daily eating patterns and their impact on health and disease. Trends Endocrinol Metab 27: 69–83. 10.1016/j.tem.2015.11.007 26706567 PMC5081399

[GAD334284SZAC235] Zeng J, Yang GY, Ying W, Kelly M, Hirai K, James TL, Swanson RA, Litt L. 2007 Pyruvate improves recovery after PARP-1-associated energy failure induced by oxidative stress in neonatal rat cerebrocortical slices. J Cereb Blood Flow Metab 27: 304–315. 10.1038/sj.jcbfm.9600335 16736046

[GAD334284SZAC236] Zerfaoui M, Suzuki Y, Naura AS, Hans CP, Nichols C, Boulares AH. 2008 Nuclear translocation of p65 NF-κB is sufficient for VCAM-1, but not ICAM-1, expression in TNF-stimulated smooth muscle cells: differential requirement for PARP-1 expression and interaction. Cell Signal 20: 186–194. 10.1016/j.cellsig.2007.10.007 17993261 PMC2278030

[GAD334284SZAC237] Zhang T, Berrocal JG, Yao J, DuMond ME, Krishnakumar R, Ruhl DD, Ryu KW, Gamble MJ, Kraus WL. 2012 Regulation of poly(ADP-ribose) polymerase-1-dependent gene expression through promoter-directed recruitment of a nuclear NAD^+^ synthase. J Biol Chem 287: 12405–12416. 10.1074/jbc.M111.304469 22334709 PMC3320990

[GAD334284SZAC238] Zhang F, Wang Y, Wang L, Luo X, Huang K, Wang C, Du M, Liu F, Luo T, Huang D, 2013 Poly(ADP-ribose) polymerase 1 is a key regulator of estrogen receptor α-dependent gene transcription. J Biol Chem 288: 11348–11357. 10.1074/jbc.M112.429134 23493398 PMC3630903

[GAD334284SZAC239] Zhang L, Zou J, Chai E, Qi Y, Zhang Y. 2014 α-Lipoic acid attenuates cardiac hypertrophy via downregulation of PARP-2 and subsequent activation of SIRT-1. Eur J Pharmacol 744: 203–210. 10.1016/j.ejphar.2014.09.037 25281201

[GAD334284SZAC240] Zhang Y, Wang C, Tian Y, Zhang F, Xu W, Li X, Shu Z, Wang Y, Huang K, Huang D. 2016 Inhibition of Poly(ADP-Ribose) polymerase-1 protects chronic alcoholic liver injury. Am J Pathol 186: 3117–3130. 10.1016/j.ajpath.2016.08.016 27746183

[GAD334284SZAC241] Zhao H, Sifakis EG, Sumida N, Millán-Arino L, Scholz BA, Svensson JP, Chen X, Ronnegren AL, de Lima CD M, Varnoosfaderani FS, 2015 PARP1- and CTCF-mediated interactions between active and repressed chromatin at the lamina promote oscillating transcription. Mol Cell 59: 984–997. 10.1016/j.molcel.2015.07.019 26321255

[GAD334284SZAC242] Zhou X, Patel D, Sen S, Shanmugam V, Sidawy A, Mishra L, Nguyen BN. 2017 Poly-ADP-ribose polymerase inhibition enhances ischemic and diabetic wound healing by promoting angiogenesis. J Vasc Surg 65: 1161–1169. 10.1016/j.jvs.2016.03.407 27288104

